# *Mycosphaerangium* and *Neomelanconium* (Cenangiaceae) are closest relatives: phylogenetic relationships, morphology and a new species

**DOI:** 10.1007/s11557-020-01630-3

**Published:** 2020-11-09

**Authors:** Hermann Voglmayr, Walter M. Jaklitsch, Salvador Tello

**Affiliations:** 1 Institute of Forest Entomology, Forest Pathology and Forest Protection, Department of Forest and Soil Sciences, BOKU-University of Natural Resources and Life Sciences, Peter-Jordan-Straße 82, 1190 Vienna, Austria; 2 Department of Botany and Biodiversity Research, University of Vienna, Rennweg 14, 1030 Wien, Austria; 3 Jaén, Spain

**Keywords:** Ascomycota, Helotiales, Incertae sedis, Leotiomycetes, *Melanconium*, Molecular phylogeny, Systematics, Taxonomy, 1 new species

## Abstract

Based on molecular phylogenetic analyses of a multigene matrix of partial nuSSU-ITS-LSU rDNA, *RPB1*, *RPB2* and *TEF1* sequences and by morphological evidence, the genus *Mycosphaerangium* is shown to be the closest relative of *Neomelanconium*, and confirmed to be a member of the Cenangiaceae (Leotiomycetes). While *Mycosphaerangium* and *Neomelanconium* share many traits like similar conidia, conidiogenesis, asci and ascospores, their apothecia differ particularly in excipular features and are therefore recognized as distinct genera. *Mycosphaerangium tiliae*, described from North America, is excluded from the genus but shown to represent the sexual morph of the European *Neomelanconium gelatosporum*, and it is therefore synonymized with the latter. Based on morphology, *Neomelanconium deightonii* is assumed to be congeneric with *Neomelanconium gelatosporum*, and it is lectotypified. *Dermatea tetraspora* and *Phaeangium magnisporum*, the basionyms of *Mycosphaerangium tetrasporum* and *M. magnisporum*, respectively, are lectotypified as well, and for *M. tetrasporum*, the asexual morph is recorded for the first time. *Mycosphaerangium quercinum* sp. nov. is described as a new species from various *Quercus* hosts in Europe, where it is shown to be widely distributed. It morphologically and ecologically closely resembles the North American *M. tetrasporum*, but differs in paraphysis and ascospore morphology and by croziers at its ascus base. The three accepted species of *Mycosphaerangium* and the two of *Neomelanconium* are described and illustrated. *Mycosphaerangium magnisporum*, *M. quercinum* and *M. tetrasporum* are recorded to be constantly associated with species of *Coryneum*, indicating a fungicolous habit, but no evidence for fungal associations has been found in *Neomelanconium deightonii* and *N. gelatosporum*.

## Introduction

The anamorph genus *Melanconium* is morphologically characterized by unicellular ellipsoid brown conidia produced in acervuli (Sutton 1964). *Melanconis*, a diaporthalean genus, was commonly accepted to be its asexual morph (Barr 1978). However, as outlined in Rossman et al. (2015) and Jaklitsch and Voglmayr (2020), the generic concept of *Melanconium* and the true identity of its generic type, *M. atrum*, are obscure, and therefore the well-defined *Melanconis* was protected over *Melanconium* (Turland et al. 2018, Appendix III). Through its history, numerous species have been described within *Melanconium*, making it a heterogeneous assemblage. Therefore, many *Melanconium* species have been subsequently transferred to other genera such as *Arthrinium*, *Greeneria* and *Harknessia* (Rossman et al. 2015). Likewise, also the large genus *Melanconis* has been shown to be polyphyletic, and it has been restricted to eight closely related species (Jaklitsch and Voglmayr 2020).

*Melanconium gelatosporum* was described by Zimmermann (1913) from *Tilia*, for which Petrak (1940) established the new genus *Neomelanconium*. Subsequently, Petrak (1954) and recently Wijayawardene et al. (2016) added two additional species, *N. deightonii* and *N. spartii*, respectively. In the original description, Zimmermann (1913) supposed diaporthalean affinities, but Petrak (1940, 1954) considered it to be the asexual morph of a member of Massariaceae (Dothideomycetes). Until recently, the systematic affiliation of the type species remained unresolved and was based solely on morphological observations. Nowadays, the genus is classified as Pezizomycotina *incertae sedis* in Index Fungorum (accessed 14 Sep. 2020). However, Crous et al. (2019) published sequence data for *N. gelatosporum* which revealed a phylogenetic affiliation with Cenangiaceae (Leotiomycetes). Also, they decided to place *N. spartii* in a new genus *Pseudomelanconium* based on morphological differences, albeit no sequence data were available for the latter. In lack of type studies and sequence data, the status of the third species, *N. deightonii*, remained unclear. No sexual morph has been published for any *Neomelanconium* species.

The sexual morph genus *Mycosphaerangium* was established by Verkley (1999) for three species originally described in the genus *Sphaerangium* (Seaver 1951), with *M. tetrasporum* as type species. This is a later homonym of the moss genus *Sphaerangium* Schimp. The three species share many traits: erumpent apothecia, dark brown to black hymenium, a lighter brown coloured excipulum, thin-walled asci with a broadly rounded apex without a visible apical apparatus, and large, subglobose to ellipsoid, dark brown ascospores surrounded by a distinct refractive, hyaline gel sheath (Verkley 1999). Due to the peculiar morphology and the lack of fresh material, Verkley (1999) classified *Mycosphaerangium* as Helotiales *incertae sedis*, which was subsequently commonly followed by other authors (e.g. Jaklitsch et al. 2016). No sequence data have yet become available for the genus, and its systematic affinities within Leotiomycetes are currently unclear.

Several collections from corticated *Quercus* twigs made by the first author revealed an apparently undescribed coelomycete resembling the genus *Neomelanconium*. Concurrently, the third author made several holomorphic collections of a discomycete on *Quercus* in Spain which showed close affinities to the North American *Mycosphaerangium tetrasporum*. A morphological comparison of the asexual morphs of all these collections revealed that they fully matched, which was further confirmed by sequence data from cultures obtained from sexual and asexual morphs. This led us to initiate a detailed study on the genera *Neomelanconium* and *Mycosphaerangium*. Fresh material of *Neomelanconium gelatosporum* was collected for morphological studies, pure culture isolation and sequencing. Type material of all three *Mycosphaerangium* and of the two *Neomelanconium* species was reviewed. In addition, multigene phylogenetic analyses were performed to solve the placement of these genera.

## Materials and methods

### Field survey, sample collection and sample sources

**Fig. 1:**
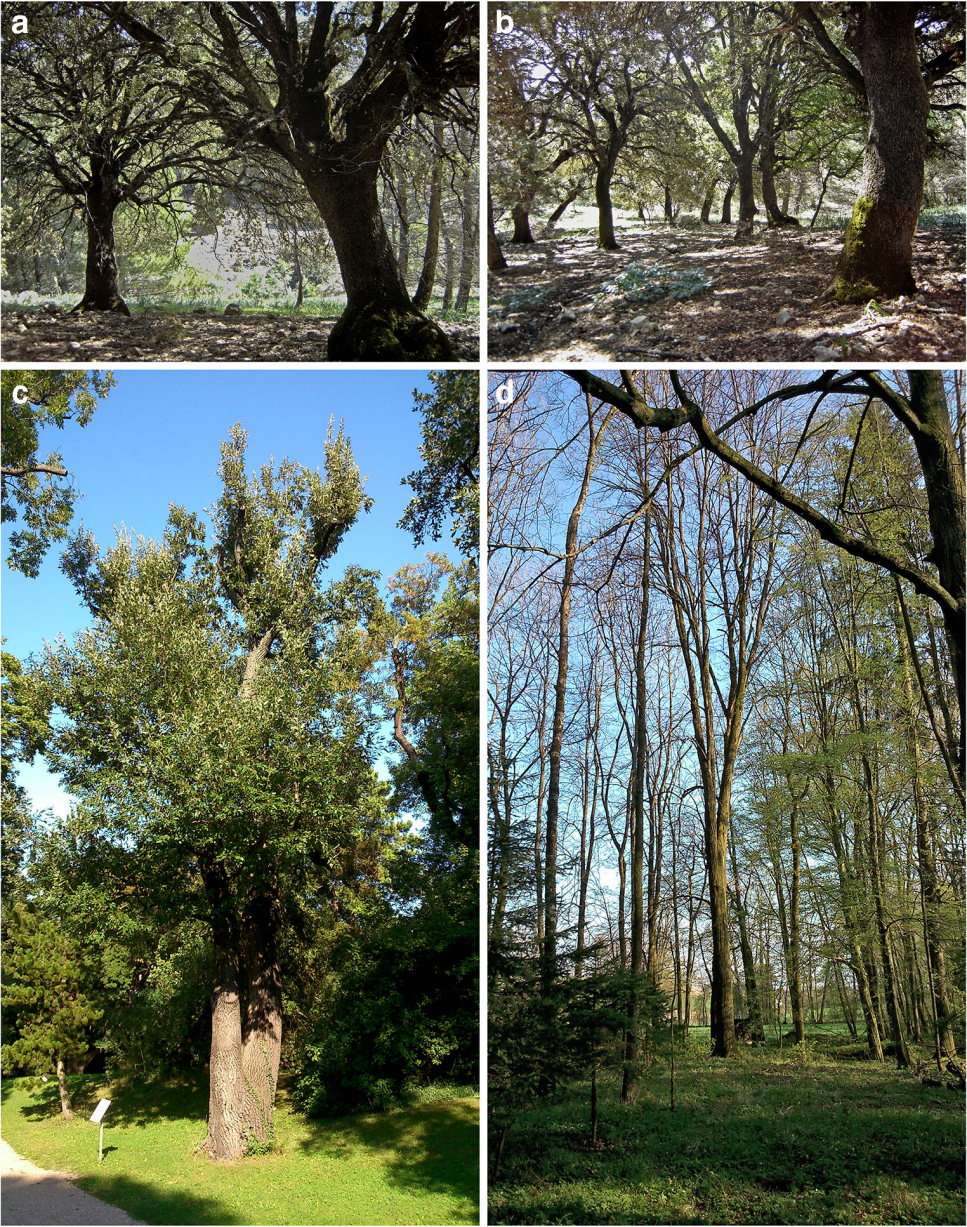
Typical habitats of *Mycosphaerangium quercinum* (**a**–**c**) and *Neomelanconium gelatosporum* (**d**)

During the period 2015–2020, recently shed or still attached dead, corticated twigs of *Quercus* spp. and *Tilia* spp. were collected, examined for the presence of fungi of interest and air dried for subsequent morphological analysis, pure culture isolation and molecular studies. Material was collected in Austria, Greece, Italy and Spain mostly in light, mixed deciduous forests, occasionally on solitary trees in alleys or parks. Typical habitats are shown in Fig. 1. After processing, specimens were deposited in the fungaria of the University of Vienna (WU) and of the Consejería de Medio Ambiente (Junta de Andalucía), Sevilla (JA-CUSSTA).

In addition to fresh collections, specimens of *Mycosphaerangium* and *Neomelanconium* spp. were received on loan from herbaria FH, GAM, GZU, NY, and W and morphologically investigated. Details of the specimens used for morphological investigations are listed in the Taxonomy section; herbarium acronyms are according to Thiers (2020), and citation of exsiccatae follows Triebel and Scholz (2020).

### Morphological observations

Stereomicroscopy photographs were captured with a Nikon SMZ 1500 stereomicroscope equipped with a Nikon DS-U2 digital camera or with a Sony Cyber-shot DSC-HX100V camera mounted in one of the oculars of a binocular lens, using a LED lamp for lighting. For certain images of ascomata, the stacking software Zerene Stacker version 1.04 (Zerene Systems LLC, Richland, WA, USA) was used.

For light microscopy, fresh collections were rehydrated and examined according to “vital taxonomy” in the sense of Baral (1992). Hand sections of ascomata and conidiomata were made using a razor blade and the following reagents were used as mounting media: tap water, 3% KOH, Congo red, Lugol’s reagent (IKI, with 3% KJ and 1% iodine) and Indian ink. Slides were examined and photographed using a Zeiss Axio Imager.A1 (Zeiss, Jena, Germany) microscope equipped with a Zeiss Axiocam 506 colour digital camera or a Canon EOS 40D camera installed on an OPTIKA B-350 microscope. Measurements were done with the NIS-Elements D v.3.0 or Zeiss ZEN Blue Edition software packages. Measurements are reported as maxima and minima in parentheses and the range representing the mean plus and minus the standard deviation of a number of measurements given in parentheses; in addition, means of measurements (Me) and of l/w ratios (Qe) are given for ascospores and conidia.

### Pure culture isolation

Mature conidiomata or apothecia were horizontally or vertically cut using a sterile razor blade and the surrounding host tissue was removed. Subsequently, the sections were transferred to a sterile drop of water on a microscope slide, torn apart with a forceps to release the conidia or the ascospores, and the suspension was pipetted on 2% corn meal agar (CMA, Sigma-Aldrich) supplemented with 2% *w*/*v* dextrose (CMD) or 2% malt extract agar (MEA) plates supplemented with 200 mg/l penicillin G and streptomycin sulphate (Sigma-Aldrich, St. Louis, MO) and incubated at room temperature. As the conidia and ascospores germinated slowly, the isolation plates were regularly checked for contaminations, which were removed. Germinated conidia or ascospores were then transferred to 2% CMD or 2% MEA plates, which were sealed with laboratory film and incubated at 22 °C. Cultures were deposited at the Westerdijk Fungal Biodiversity Centre, Utrecht, The Netherlands (CBS culture collection).

### DNA extraction, PCR and sequencing

Growth of liquid cultures and extraction of genomic DNA was done according to Voglmayr and Jaklitsch (2011), using the DNeasy Plant Mini Kit (QIAgen GmbH, Hilden, Germany). Six loci (ITS, LSU, and SSU rDNA, *RPB1*, *RPB2*, *TEF1*) were amplified. For details on the primers and annealing temperatures used for PCR and sequencing, see Table 1. PCR products were purified using an enzymaticPCR cleanup (Werle et al. 1994) as described in Voglmayr andJaklitsch (2008). DNA was cycle-sequenced using the ABI PRISM Big Dye Terminator Cycle Sequencing Ready Reaction Kit v. 3.1 (Applied Biosystems, Warrington) and the PCR primers; in addition, for some loci, internal primers were used (see Table 1). Sequencing was performed on an automated DNA sequencer (ABI 3730xl Genetic Analyzer, Applied Biosystems).

**Table 1:** Primers used to amplify and sequence the nuclear internal transcribed spacer (ITS), large subunit (LSU) and small subunit (SSU) rDNA regions, the RNA polymerase II largest (*RPB1*) and second largest (*RPB2*) subunit genes and the translation elongation factor 1-α (*TEF1*) gene

Gene	Primer	Sequence (5′–3′)	Direction	Annealing *t* (°C)	Reference
ITS-LSU					
	V9G	TTAAGTCCCTGCCCTTTGTA	Forward	55	Hoog and Gerrits van den Ende (1998)
	LR5	TACTTGAAGGAACCCTTACC	Reverse		Vilgalys and Hester (1990)
	LR2R-A^1^	CAGAGACCGATAGCGCAC	Forward		Voglmayr et al. (2012)
	LR3^1^	CCGTGTTTCAAGACGGG	Reverse		Vilgalys and Hester (1990)
	ITS4^1^	TCCTCCGCTTATTGATATGC	Reverse		White et al. (1990)
SSU					
	SL1	TGGTTGATCCTGCCAGTA	Forward	53	Voglmayr and Jaklitsch (2011)
	NS24mod	GAAACCTTGTTACGACTTTTAC	Reverse		White et al. (1990)
	NS1088^1^	TGATTTCTCGTAAGGTGCCG	Reverse		Kauff and Lutzoni (2002)
*RPB1*					
	RPB1-Af	GARTGYCCDGGDCAYTTYGG	Forward	55	Stiller and Hall (1997)
	RPB1-6R1asc	ATGACCCATCATRGAYTCCTTRTG	Reverse		Hofstetter et al. (2007)
*RPB2*					
	dRPB2-5f	GAYACNGAYGAYCGWGAYCAYTTYGG	Forward	52	Voglmayr et al. (2016)
	dRPB2-7r	AANCCCATDGCYTGYTTDCCCAT	Reverse		Voglmayr et al. (2016)
*TEF1*					
	EF1-728F	CATCGAGAAGTTCGAGAAGG	Forward	55	Carbone and Kohn (1999)
	EF1-2218R	TACTTGAAGGAACCCTTACC	Reverse		Carbone and Kohn (1999)
	TEF1_INTF^1^	CCGTGAYTTCATCAAGAACATG	Forward		Jaklitsch (2009)
	TEF1_INTF	CCGTGAYTTCATCAAGAACATG	Forward	55	Jaklitsch (2009)
	EF1-2218R	TACTTGAAGGAACCCTTACC	Reverse		Carbone and Kohn (1999)

^1^Internal primers used only for sequencing

### Phylogenetic analyses

For phylogenetic analyses, a matrix of aligned nucleotide sequences from six different phylogenetic markers (ITS, LSU, and SSU rDNA, *RPB1*, *RPB2*, *TEF1*) was produced. Sequences obtained in the present study were aligned to selected GenBank sequences of Cenangiaceae, Rutstroemiaceae and Sclerotiniaceae from Pärtel et al. (2017) and Johnston et al. (2019) and complemented with a few additions from GenBank. Five species of *Chlorociboria* (*Chlorociboriaceae*) were selected as outgroup according to Johnston et al. (2019). Sequences were aligned with the server versions of MAFFT (Katoh et al. 2002; http://www.ebi.ac.uk/Tools/mafft (Madeira et al. 2019), https://mafft.cbrc.jp/alignment/server (Katoh et al. 2019)) and subsequently checked, trimmed and refined using BioEdit version v. 7.0.9.0 (Hall 1999), removing excessive leading and trailing gap regions and adjusting the protein-coding gene alignments according to the correct amino acid reading frame. The combined sequence matrix contained 6677 nucleotide positions (555 from ITS, 1299 from LSU, 1633 from SSU, 1104 from *RPB1*, 1076 from *RPB2*, 1010 from *TEF1*). GenBank accession numbers of the sequences included in the phylogenetic analyses are given in Table 2; the species classification follows Galán et al. (2015), Johnston et al. (2019) and Perić et al. (2019).

**Table 2:** Isolates and accession numbers used in the phylogenetic analyses. Taxa with quoted genus names are generically misplaced (polyphyletic) but not yet formally recombined. Isolates/sequences in bold were isolated/sequenced in the present study

Taxon	Voucher/culture	SSU	ITS	LSU	*RPB1*	*RPB2*	*TEF1*
*Bicornispora seditiosa*	AH44702	-	KF499362	KF499362	-	**MW001932**	**MW001933**
*Botrytis cinerea*	OSC 100012	AY544695	DQ491491	AY544651	DQ471116	DQ247786	DQ471045
*Calycellinopsis xishuangbanna*	HMAS 187063	GU936124	-	KR094163	MH729338	MH729345	-
*Cenangiopsis alpestris*	B.P.Dgf = C7D-06-05-14	KX090891	LT158470	KX090839	KX090786	KX090738	-
*Cenangiopsis quercicola*	TAAM 178677	KX090862	LT158425	KX090811	KX090760	KX090713	KX090663
*Cenangiopsis junipericola*	B.P.Dgf = C7D-02-07-14(2)	KX090890	KX090900	KX090838	KX090785	KX090737	-
*‘Cenangium’ acuum*	TAAM 198515 = H.B. 9325b	KX090873	LT158439	KX090822	KX090767	KX090720	KX090674
*Cenangium ferruginosum*	TAAM 198451	KX090892	LT158471	KX090840	-	KX090739	-
*Chlorencoelia torta*	M-0281036 = H.B.8415	-	LT158424	KX090810	KX090759	-	-
*Chlorencoelia versiformis*	TU 107606	KX090894		-	KX090788	KX090740	KX090692
*Chlorociboria aeruginascens*	TAAM 198512 = H.B.7852	-	LT158419	-	KX090752	KX090706	KX090657
*Chlorociboria aeruginella*	TAAM 198514 = H.B. 9450	KX090875	MH752067	-	KX090769	KX090722	KX090676
*Chlorociboria aeruginosa*	OSC 100056	AY544713	DQ491501	AY544669	DQ471125	DQ470886	DQ471053
*Chlorociboria glauca*	TAAM 198458 = H.B.9232	KX090872	-	KX090821	KX090766	-	KX090673
*Chlorciboria halonata*	ICMP 15625	JN939862	AY755355	JN939933	JN985211	JN985511	-
*Ciboria viridifusca*	TAAM 165962	KX090863	LT158429	KX090812	-	-	-
*Crumenulopsis sororia*	TU 104504	-	-	KX090826	-	KX090725	-
*Dumontinia tuberosa*	TU 109263	KX090897	-	KX090843	KX090792	–	KX090697
*Encoelia furfuracea*	TAAM 165633	KX090850	LT158416	KX090798	KX090749	KX090701	KX090653
*Heyderia abietis*	TAAM 165961	KX090845	LT158426	-	KX090747	KX090699	KX090650
*Heyderia pusilla*	TU 104257	KX090865	LT158430	-	KX090762	KX090715	KX090665
*‘Lambertella’ brunneola*	TNS-F-44244	LC434593	LC425046	MWLR Datastore^1^	LC431692	LC431720	-
*Lambertella corni-maris*	TNS-F-40083	LC434562	AB926069	AB926139	-	AB926184	-
*Lambertella palmeri*	AH 7655	-	KF499365	KF499365	-	-	-
*Lambertella pyrolae*	TNS-F-40132	LC434565	AB926081	AB926164	-	AB926209	-
*‘Lambertella’ subrenispora*	CBS 811.85	DQ471030	MH861915	DQ470978	DQ471176	DQ470930	DQ471101
*Meria laricis*	CBS 298.52	DQ471002	KT225534	DQ470954	DQ471146	DQ470904	DQ842026
*Moellerodiscus lentus*	HMAS 275557	MH729334	KU668566	MH729337	MH729343	MH729344	-
*Moellerodiscus pinicola*	TNS-F-40115	LC434564	AB926078	AB926162	-	AB926192	-
*Monilinia laxa*	CBS 122031	AY544714	-	AY544670	FJ238425	DQ470889	DQ471057
*Mycosphaerangium quercinum*	**D135 = CBS 144229**	**MT952888**	**MT952893**	**MT952893**	**MT996193**	**MT996196**	-
*Mycosphaerangium quercinum*	**EXT1**	-	**MT952892**	**MT952892**	-	-	**MT996200**
*Mycosphaerangium quercinum*	**MYT = CBS 147011**	-	**MT952891**	**MT952891**	**MT996194**	**MT996197**	-
*Neomelanconium gelatosporum*	CBS 144985	-	MN313810	MN317291	MN313871	-	-
*Neomelanconium gelatosporum*	CPC 31127	-	MN313811	MN317292	MN313870	-	-
*Neomelanconium gelatosporum*	**NG = CBS 143625**	**MT952887**	**MT952889**	**MT952889**	**MT996195**	**MT996198**	**MT996201**
*Neomelanconium gelatosporum*	**NG1 = CBS 143626**	-	**MT952890**	**MT952890**	-	**MT996199**	**MT996202**
*Piceomphale bulgarioides*	TAAM 165289	KX090848	LT158483	KX090797	-	KX090700	-
*Poculum pseudosydowianum*	TNS-F-40071	LC434561	AB904505	AB926136	-	AB926182	-
*‘Pseudopeziza’ colensoi*	PDD 112240	-	MH921874	MH985297	MH986706	MH986705	-
*Pycnopeziza sejournei*	C- F(J.H.P. 11,054)	KX090878	LT158443	KX090827	KX090772	KX090726	KX090679
*Rutstroemia bolaris*	TU 104236	KX138402	LT158432	KX138406	-	-	KX138397
*Rutstroemia firma*	TU 104481	KX090881	LT158450	KX090832	KX090774	KX090731	KX090684
*Rutstroemia johnstonii*	C-F 28009	KX090884	LT158454	-	KX090777	KX090733	KX090687
*Rutstroemia juniperi*	O 212357	KX090871	-	KX090820	-	-	KX090672
*Rutstroemia luteovirescens*	TU 104450	-	LT158431	KX090814	KX090763	KX090716	KX090666
*Rutstroemia tiliacea*	TAAM 132844 = H.B.6734	KX090860	LT158423	KX090808	KX090757	KX090711	KX090661
*Sclerencoelia fraxinicola*	M-0281054 = H.B.5714	KX090857	LT158420	KX090805	KX090755	KX090708	KX090659
*Sclerencoelia pruinosa*	NY 02533482	KX090888	LT158462	-	KX090781	KX090735	-
*Sclerotinia sclerotiorum*	CBS 499.50	DQ471013	MH856725	DQ470965	-	DQ470916	DQ471086
*Trochila craterium*	BPI 880163	KX090886	-	-	KX090779	-	-
*Trochila laurocerasi*	BPI 879818	KX090887	LT158460	KX090835	KX090780	KX090734	KX090689
*Velutarina rufo-olivacea*	TU 104503	KX090877	-	KX090825	KX090771	KX090724	KX090678

^1^Obtained from Manaaki Whenua-Landcare Research Datastore (https://datastore.landcareresearch.co.nz/ne/dataset/leotiomycete-phylogeny-2018)

Maximum likelihood (ML) analyses were performed with RAxML (Stamatakis 2006) as implemented in raxmlGUI 1.3 (Silvestro and Michalak 2012), using the ML + rapid bootstrap setting and the GTRGAMMA substitution model with 1000 bootstrap replicates. The matrix was partitioned for the individual gene regions, with separate substitution model parameters implemented.

Maximum parsimony (MP) analyses were performed with PAUP v. 4.0a167 (Swofford 2002), using 1000 replicates of heuristic search with random addition of sequences and subsequent TBR branch swapping (MULTREES option in effect, steepest descent option not in effect). All molecular characters were unordered and given equal weight; analyses were performed with gaps treated as missing data; the COLLAPSE command was set to NO. Bootstrap analysis with 1000 replicates was performed in the same way, but using 10 rounds of random sequence addition and subsequent TBR branch swapping during each bootstrap replicate.

Bootstrap support below 70% was considered low, between 70 and 90% moderate, above 90% high and 100% maximum.

## Results

### Culture characteristics

**Fig. 2:**
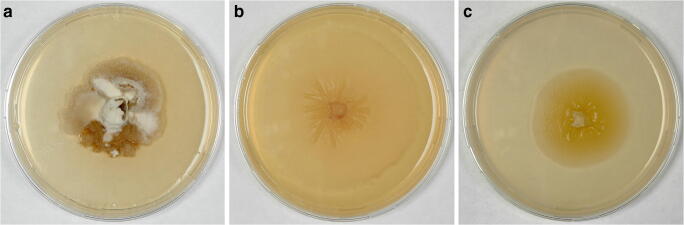
Cultures (22 °C). (**a**) *Mycosphaerangium quercinum* D135 on CMD after 3 months. (**b, c**) *Neomelanconium gelatosporum* NG on MEA (**b**) and CMD (**c**) after 2 months

Culture images of *Mycosphaerangium quercinum* and *Neomelanconium gelatosporum* grown on 2% CMD and 2% MEA are shown in Fig. 2. Culture descriptions are given under the respective species.

### Molecular phylogeny

**Fig. 3:**
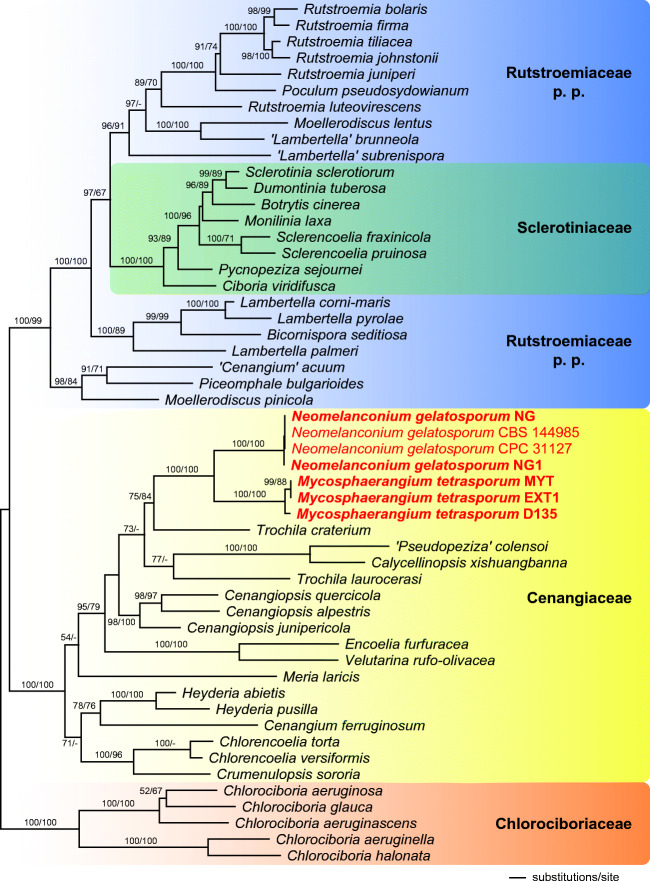
Phylogram of the best ML trees (−lnL = 54940.906) revealed by RAxML from an analysis of the combined SSU–ITS–LSU–*RPB1*–*RPB2*–*TEF1* matrix of selected Helotiales, showing the phylogenetic position of *Mycosphaerangium* and *Neomelanconium* within Cenangiaceae. Strains in bold were sequenced in the current study. ML and MP bootstrap supports above 50% are given at the first and second positions, respectively, above or below the branches

Of the 6677 nucleotide positions, 1882 were parsimony informative (238 from ITS, 198 from LSU, 140 from SSU, 526 from *RPB1*, 493 from *RPB2* and 287 from *TEF1*). The parsimony analyses revealed 1 MP tree 10,574 steps long (not shown). The best tree revealed by RAxML (− ln = 54,940.906) is shown as phylogram in Fig. 3. Except for a few deeper nodes, topologies of the ML tree were largely compatible with the MP tree. Tree topologies of our phylogenetic analyses agree well with those of Johnston et al. (2019). In the phylogenetic analyses, the Cenangiaceae clade and the Rutstroemiaceae-Sclerotiniaceae clade receive maximum support. Within the latter, the Sclerotiniaceae are resolved as a highly supported clade, but they are embedded within a paraphyletic Rutstroemiaceae, which consists of three highly supported subclades. Within Cenangiaceae, *Mycosphaerangium quercinum* and *Neomelanconium gelatosporum* are sister species with maximum support, and the sister group relationship of the *Mycosphaerangium-Neomelanconium* clade with *Trochila craterium* is moderately supported. As previously reported (e.g. Pärtel et al. 2017, Johnston et al. 2019), several genera like *Cenangium*, *Lambertella*, *Moellerodiscus* and *Trochila* are polyphyletic, indicating that the morphological characters currently used for generic circumscription do not reflect phylogenetic relationships, necessitating additional morphological and molecular investigations to reach more appropriate generic concepts within Helotiales.

### Taxonomy

***Mycosphaerangium*** Verkley, Stud. Mycol. 44: 156. 1999.

*= Sphaerangium* Seaver, North American Cup-fungi, (Inoperculates) (New York): 308. 1951. Nom. illegit., Art. 53.1.

*Type species*: *Mycosphaerangium tetrasporum* (Ellis) Verkley.

*Sexual morph* apothecial, erumpent through bark, solitary or in groups, sessile to subsessile; basal stroma absent. *Recpetacle* paler than the disc, beige, orange to dark brown, with raised, rim-like margins, curved inward when dry. *Disc* circular, elliptic to irregularly polyangular, concave to plane, dark brown to black, rough. *Paraphyses* embedded in hymenial gel, longer than the asci, filiform, septate, simple or branched, sometimes anastomosing, with hyaline to brownish, slightly to distinctly swollen or irregularly sinuous-knobby tips, refractive vacuolar bodies (VBs) absent. *Asci* cylindrical-clavate, thin-walled, unitunicate, with broadly rounded to subtruncate apex without a distinct apical apparatus and opening by an apical rupture, with 4 uniseriately arranged ascospores. *Ascospores* dark brown, aseptate, subglobose or ellipsoid, with a thick, dark brown, minutely to distinctly warted wall, surrounded by a prominent hyaline gelatinous sheath rapidly expanding in water.

*Asexual morph* acervular, usually conspicuous due to black, glossy, effused conidial masses, associated with conidiomata or stromata of *Coryneum* spp. *Conidiomata* subperidermal, not to slightly elevating the bark, solitary, scattered, lenticular, with thin, whitish pseudoparenchymatous base. *Conidiophores* emerging from a pseudoparenchymatous base, reduced to the conidiogenous cells. *Conidiogenous cells* percurrent, smooth, cylindrical to filiform, hyaline, commonly turning yellowish-brown with age, with a single, often indistinct annellation. *Conidia* dark brown, aseptate, subglobose, broadly ellipsoid, ovoid to pyriform, apex broadly rounded, base truncate, thick-walled, guttulate, usually with one large and numerous small guttules, with smooth to finely verruculose wall, surrounded by a prominent hyaline gelatinous sheath rapidly expanding in water.

*Notes*: No asexual morph has been known for *Mycosphaerangium* until this study, a connection which is here confirmed by morphological and molecular phylogenetic data. The three species here accepted within the genus, *M. magnisporum*, *M. quercinum* and *M. tetrasporum*, are closely associated with *Coryneum* conidiomata or stromata, indicating a fungicolous ecology. *Mycosphaerangium tiliae* is removed from the genus based on apothecial morphology, differing by 8-spored asci and by marginal, inversely stellate excipular teeth with a textura prismatica-porrecta and covered by an external layer of white crystals; *M. tiliae* is synonymized with *Neomelanconium gelatosporum* (see below).

**Fig. 4:**
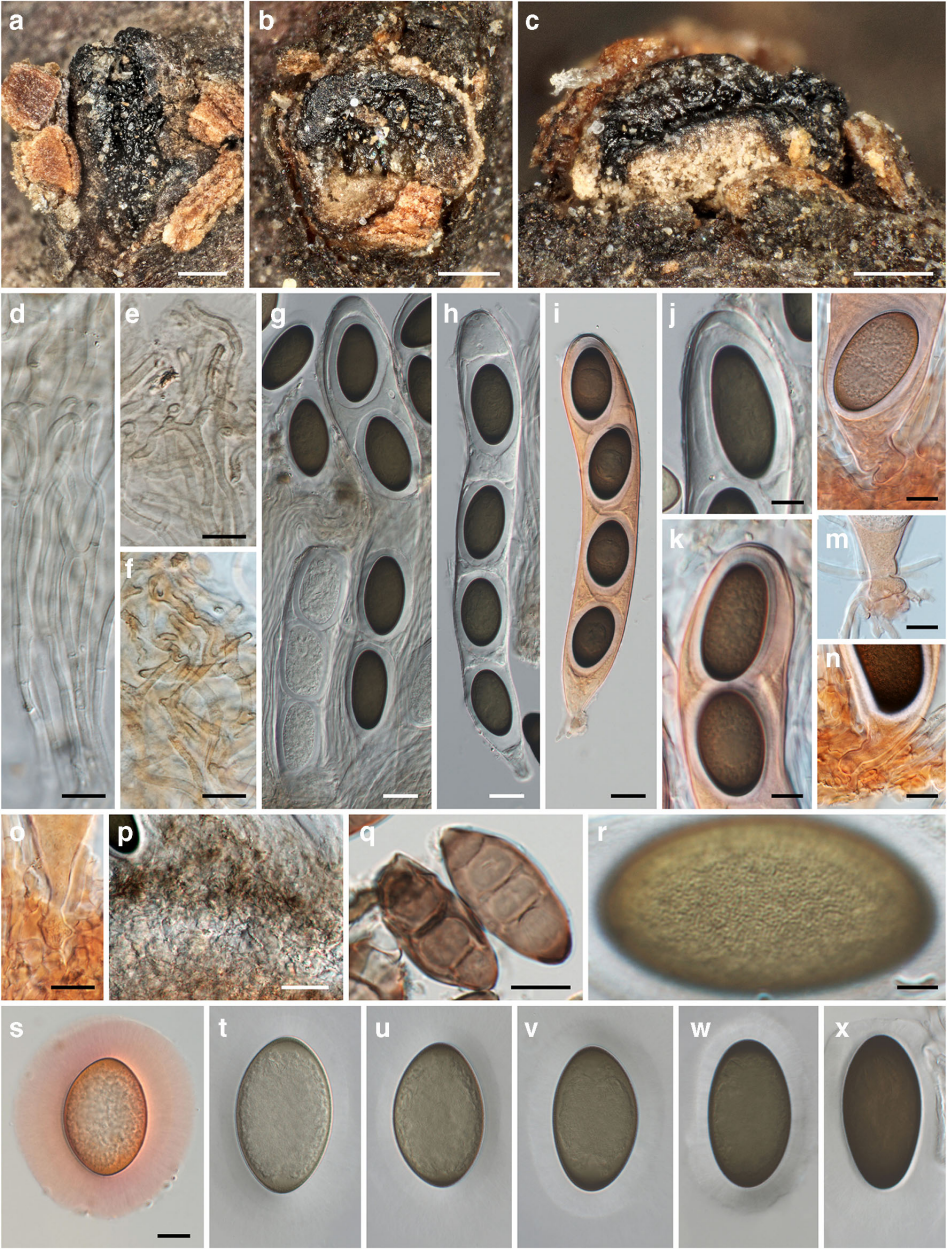
*Mycosphaerangium magnisporum*, sexual morph ((**a**) NY 02975297, isotype; (**b–e, g, h, j, p, r, t–x**) NY 02975298, lectotype; (**f, i, k–o, q, s**) FH 00965165, isotype). (**a, b**) Apothecia in face view. (**c**) Apothecium in side view. (**d**) Septate anastomosing paraphyses. (**e**, **f**) Branched paraphysis tips with brown, finely verruculose encrustation. (**g**–**i**) Asci with four ascospores surrounded by thick gelatinous sheath. (**j**, **k**) Ascus apices. (**l**–**o**) Ascus bases with simple septa. (**p**) Subhymenium. (**q**) Conidia of *Coryneum betulinum* associated with apothecia. (**r**–**x**) Ascospores with swelling gelatinous sheath (in (**r**) showing fine verrucae on inner ascospore wall, (**s**) immature). All mounts dead and in 3% KOH, except (**f, i, k–o, s**) in Congo red. Scale bars: (**a–c**) 200 μm; (**d–f, j–o, q, s–x**) 10 μm; (**g–i, p**) 20 μm; (**r**) 5 μm

***Mycosphaerangium magnisporum*** (E.K. Cash) Verkley, Stud. Mycol. 44: 157. 1999. Figure 4.

*Basionym*: *Phaeangium magnisporum* E.K. Cash, J. Wash. Acad. Sci. 30: 304. 1940.

≡ *Sphaerangium magnisporum* (E.K. Cash) Seaver, North American Cup-fungi, (Inoperculates) (New York): 309. 1951.

*Sexual morph*: *Apothecia* 1–1.5-mm diam, erumpent from bark, solitary or in groups of 2–3, sessile, without a basal stroma, circular to elliptic, plane to concave, often compressed and contorted, black. *Receptacle* finely scaly with the outer surface beige to medium brown, margin concolourous and slightly raised. *Hymenium* roughened, dark brown to black. *Subhymenium* made of subhyaline to brownish textura intricata. *Paraphyses* ca. 250–300 μm long, 1.5–3 μm wide, cylindrical, hyaline, septate, branched, slightly longer than the asci, with branched, densely interwoven, not to slightly inflated tips with brownish, finely verruculose encrustation. *Asci* (195–)210–275(–300) × (30.5–)34–41(–44) μm (*n* = 27), cylindrical, unitunicate, with rounded to subtruncate apex, inamyloid, without visible apical apparatus, containing 4 uniseriately arranged ascospores, base tapering, with simple septum. *Ascospore dehiscence* unknown. *Ascospores* (37–)40.5–46(–50) × (18–)21–25(–29) μm, l/w = (1.5–)1.7–2.1(–2.6), Me = 43 × 23 μm, Qe = 1.9 (*n* = 103), ellipsoid, first hyaline, turning medium to dark brown when mature, with darker brown ends, containing a large globose oil drop, wall appearing smooth in profile view, inner wall finely verruculose, with a prominent refractive gelatinous sheath widely expanded after release.

*Asexual morph*: unknown.

*Habitat*: on dead corticated branches of *Betula nigra*, associated with effete conidiomata of *Coryneum betulinum*.

*Distribution*: only known from GA, southeastern USA.

*Typification*: USA, GA, Clarke, Athens, University of Georgia, Agricultural Campus, in bark of *Betula nigra*, 2 Mar. 1939, leg. G.E. Thompson & J.H. Miller (NY 02975298, lectotype here designated; MBT393680; isotypes NY 02975297, BPI 665743, FH 00965165; sexual morph).

*Additional specimen examined*: USA, GA, Clarke, Athens, University of Georgia, Agricultural Campus, in bark of *Betula nigra*, 14 Feb. 1939, leg. G.E. Thompson (GAM 00010414; depauperate).

*Notes*: *Mycosphaerangium magnisporum* is so far only known from two sites in GA, USA (see https://mycoportal.org/). The type collection, investigated and described by Edith K. Cash at the USDA, Beltsville, USA, was distributed to several herbaria in the USA, of which we here select the copy NY 02975298 as lectotype. In addition, a specimen from the same locality but with an earlier collection date was received from GAM; although it is marked as co-type, it is not mentioned in the protologue and therefore does not qualify for a type. The specimens seen contain only few ascomata and are in rather poor condition. Most apothecia seem to have fallen off which is already mentioned in the original description (Cash 1940). To save material, microscopy of the specimens was kept to a minimum, and some characters like excipulum morphology could not be documented. No conidiomata were seen on the specimens. Like in *M. quercinum* and *M. tetrasporum*, the apothecia of *M. magnisporum* are associated with a *Coryneum* species; it could be identified as *C. betulinum* (Fig. 4q). Despite the lack of sequence data and conidiomata, morphology and ecology leave no doubt about its affiliation with *Mycosphaerangium*. It differs from the other species by distinctly longer, ellipsoid ascospores with darker brown ends and a different host.

**Fig. 5:**
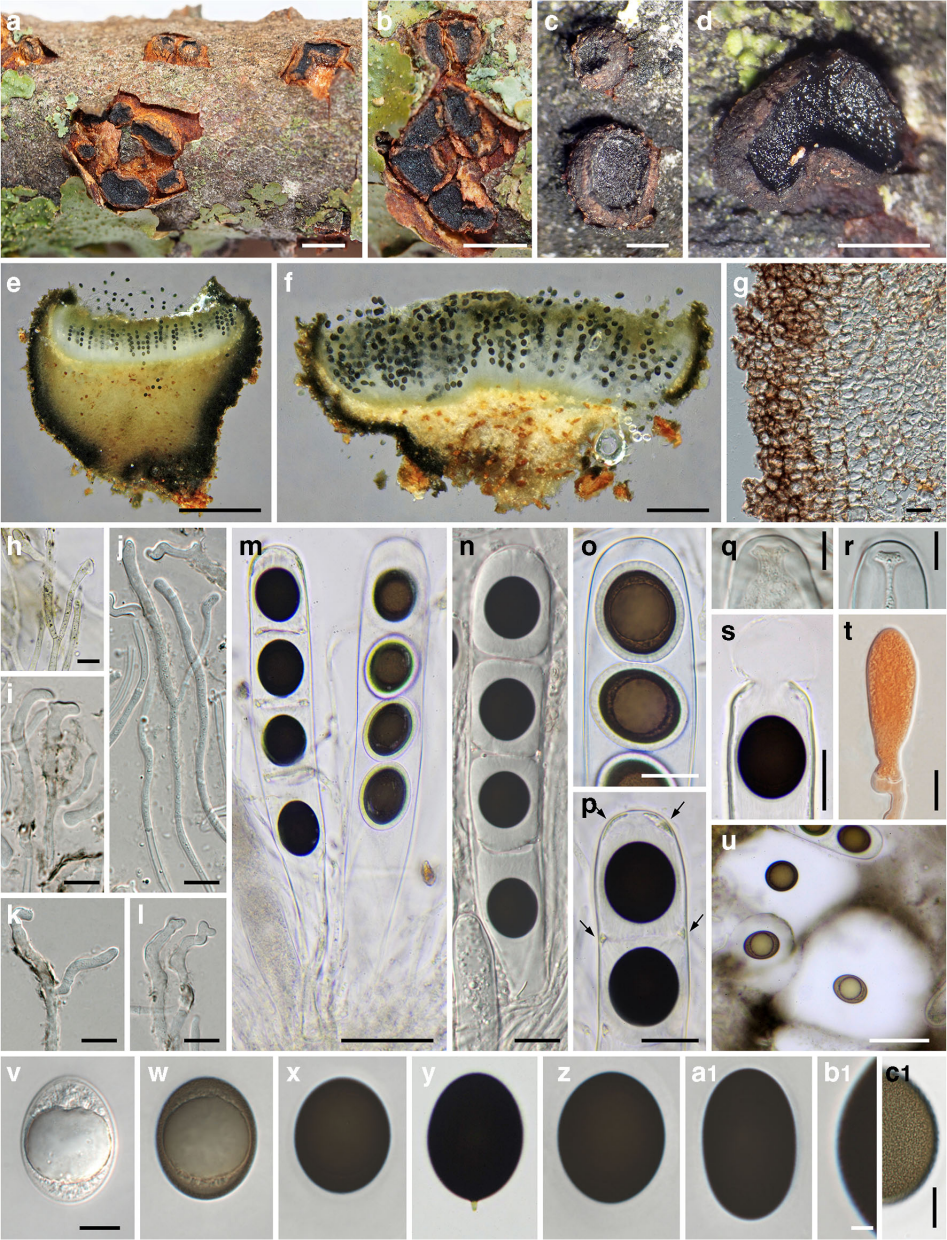
*Mycosphaerangium quercinum*, sexual morph ((**a**, **b**) S.T.13081703; (**c–f, m–p, s, u**) WU 40038; (**g–l, q, r, t, v–x, z–c1**) WU 40027, holotype; (**y**) JA-CUSSTA 8297). (**a**–**d**) Fresh apothecia in face view. (**e**, **f**) Apothecia in section. (**g**) Marginal excipulum (dead). (**h**–**l**) Septate paraphyses with irregularly sinuous-knobby tips and brownish encrustation ((**h**) vital, (**i**–**l**) dead). (**m**, **n**) Asci with four ascospores surrounded by thick gelatinous sheath ((**m**) vital, (**n**) dead). (**o**–**s**) Ascus apices ((**o**, **p**) vital, (**q**, **r**) dead, (**s**) showing apical rupture; arrows in (**p**) denoting membrane residues of broken expanded gel sheath). (**t**) Young ascus with basal crozier. (**u–c1**) Ascospores (in (**u)** showing widely expanded gelatinous sheath; in (**y**) with basal apiculus, (**b1, c1**) finely verruculose contours and wall in face view; (**u–b1**) vital, (**c1**) dead). All in water, except (**g, i–l, n, q, r**) in 3% KOH, (**t**) in Congo red, (**u**) in Indian ink. Scale bars: (**a, b**) 2 mm; (**c, d**) 1 mm; (**e**) 500 μm; (**f**) 200 μm; (**g, n–p, s**) 20 μm; (**h–l, q, r, t, v–a1, c1**) 10 μm; (**m, u**) 50 μm; (**b1**) 2 μm

**Fig. 6:**
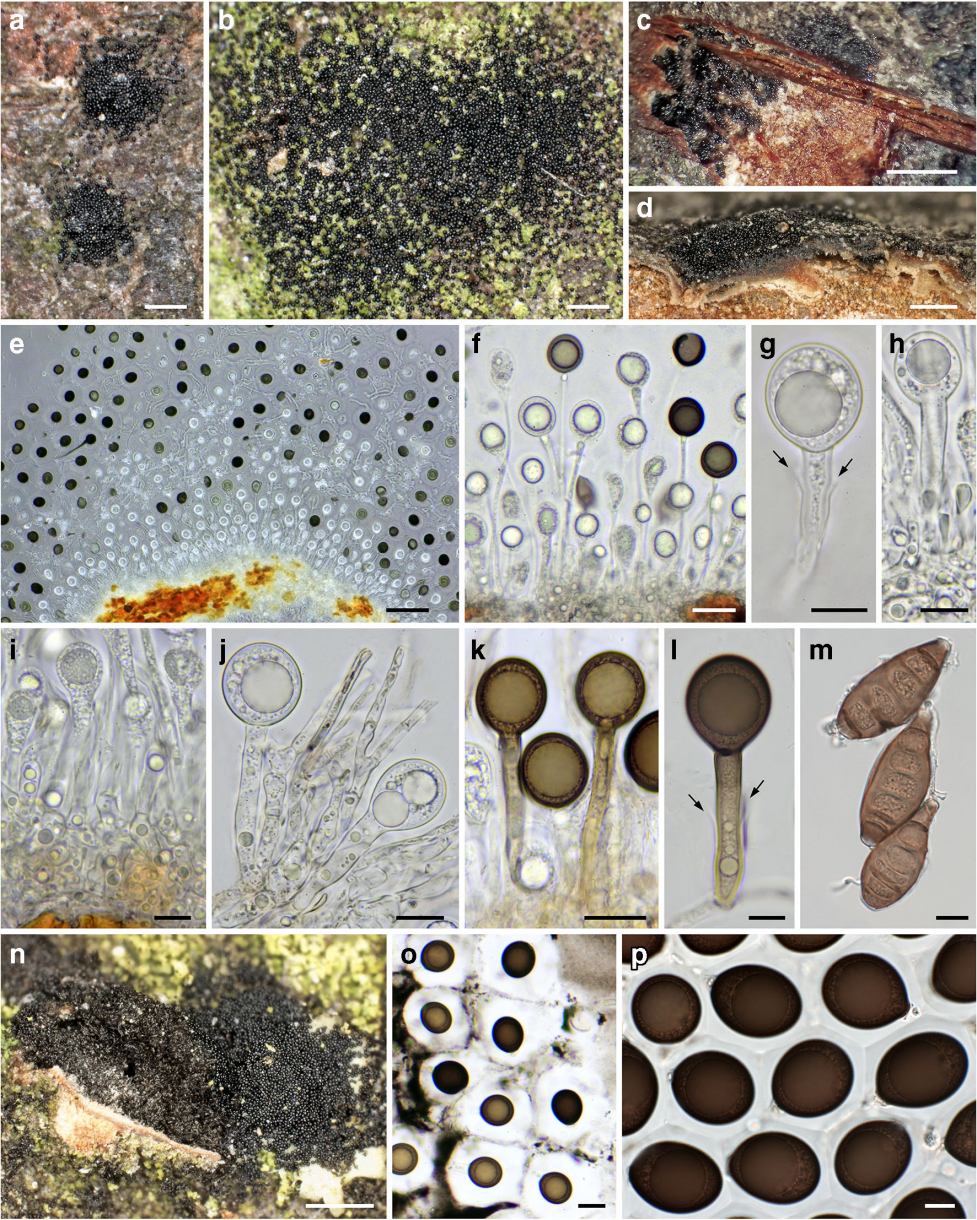
*Mycosphaerangium quercinum*, asexual morph ((**a**, **c**, **e**–**l**, **n**) WU 40027; (**b**) WU 40030; (**d, o**) WU 40031; (**m**) WU 40032; (**p**) WU 40028). (**a, b**) Conidiomata with dispersed, glossy conidia in face view. (**c**) Conidioma in transverse section, (**d**, **e**) Conidioma in vertical section. (**f**–**l**) Conidiophores, conidiogenous cells and immature (**f**–**j**) and mature (**k**, **l**) conidia; arrows denoting single annellations of the conidiogenous cells. (**m**, **n**) Conidia (**m**) and conidioma (**n**) of *Coryneum* cf. *depressum* associated with conidioma of *M. quercinum*. (**o**, **p**) Conidia with expanded gelatinous sheaths. All in water; (**m**) dead, all others vital. Scale bars: (**a–d**) 200 μm; (**e**) 100 μm; (**f**) 30 μm, (**g–j, l, m, p**) 10 μm; (**k, o**) 20 μm; (**n**) 500 μm

***Mycosphaerangium quercinum*** Voglmayr, Jaklitsch & Tello, sp. nov. Figures 5 and 6.

MycoBank: MB 836870.

*Etymology*: referring to its *Quercus* hosts.

*Diagnosis*: *Mycosphaerangium quercinum* is distinguished from the other species of the genus mainly by irregularly sinuous-knobby paraphysis tips encrusted by amorphous, olive to reddish brown plaques.

*Sexual morph*: *Apothecia* (0.4–)0.9–2.5(–4)-mm diam (*n* = 82), erumpent from bark, solitary or in groups of 2–5, sessile, without a basal stroma, first closed, later opening to form circular to irregular concave discs. *Receptacle* scaly with the outer surface dark orange- to red-brown, margin raised, slightly paler, smooth to slightly lacerate. *Hymenium* roughened, dark brown to black, sometimes with light brown scales at the margins. *Ectal excipulum* consisting of a (41–)45–150(–165) μm thick layer, narrower at the margin, consisting of brown textura globulosa-angularis, cells at margin (4.5–)8.5–15(–21)-μm diam (*n* = 105). *Medullary excipulum* up to 700 μm thick at the base, consisting of yellowish brown to subhyaline textura globulosa-angularis, intermingled with some textura prismatica, with oil drops. *Subhymenium* made of hyaline textura intricata of (2.5–)3.2–5.3(–8) μm (*n* = 57) wide hyphae with oil contents. *Paraphyses* ca. 165–205 μm long, (1.7–)2.2–3.3 μm wide, cylindrical, hyaline, septate, branched, with lipid content at least in their lower thirds, with irregularly sinuous-knobby tips widened to 3–6 μm, paraphysis tips encrusted by amorphous, olive to reddish brown plaques, refractive vacuolar bodies (VBs) absent. *Asci* (179–)215–273(–311) × (29.5–)31.5–37(–44) μm (*n* = 88), cylindrical-clavate, unitunicate, with rounded to subtruncate apex, inamyloid, without visible apical apparatus and with spore discharge by apical rupture (Fig. 5s), containing 4 uniseriately arranged ascospores, base tapering, with crozier (Fig. 5t). *Ascospores* (25–)28.5–33(–37) × (19–)22.3–25(–27.7) μm, l/w = (1.1–)1.2–1.4(–1.8), Me = 30.6 × 23.7 μm, Qe = 1.3 (*n* = 259), subglobose to ellipsoid, rarely with an apiculus on one end to 2.8 μm long (Fig. 5y), first hyaline, turning pale brown and finally dark brown to nearly black when mature, containing a large globose oil drop (17.5–)18.5–20.7(–22.8)-μm diam (*n* = 67) and numerous small ones, with (0.9–)1.1–1.6(−1.7) μm thick, finely verruculose walls that appear smooth in profile view, with a (2–)2.5–3.5(–4) μm thick (*n* = 66) gelatinous sheath (Fig. 5o) surrounded by a delicate membrane breaking upon maturation or by mechanical pressure, leaving its residues among the mass of swollen sheaths or at the ascus wall (Fig. 5p); sheath expanding around the spores forming longitudinal striae inside the ascus (Fig. 5o, p), widely expanded in released spores (Fig. 5u).

*Asexual morph* on the natural substrate: *Conidiomata* acervular, subperidermal, scattered, scarcely raising the bark, becoming visible in the dry state by black irregular spots of distinctly glossy conidia and sometimes small bumps in the bark after the discharge of the conidia through bark fissures. In the moist state, a gelatinous hyaline mass with small black conidial dots appearing. *Conidiomata* consisting of a whitish to pale yellowish, compact, more or less pseudoparenchymatous, (17–)19–38(–50)-μm-thick base; cells hyaline, septate, elongate, intertwined, thin-walled, (2.5–)3.5–8(–8.7) μm wide, intermingled with some globose cells, containing numerous oil drops, giving rise to the conidiophores. *Conidiophores* reduced to conidiogenous cells. *Conidiogenous cells* percurrent, cylindrical, filiform, slightly enlarged apically, (13–)34–117(–225) × (2.3–)4.2–6.8(−9) μm (*n* = 135), non-septate, hyaline, turning brown with age; with only a single annellation and conidium on top. *Conidia* (22.5–)27–34(–41) × (20–)23.5–28(–33) μm, l/w = (1.0–)1.1–1.3(–1.5), Me = 30.4 × 25.8 μm, Qe = 1.18 (*n* = 223), globose to ellipsoid, with (1.1–)1.2–1.7(–1.8)-μm-thick wall, first hyaline, turning brown to nearly black, containing a large globose oil drop (14–)18–21(–23.5)-μm diam (*n* = 147) and numerous small ones, finely verruculose, with a (7–)10.5–18.5(–24.5)-μm-thick gelatinous sheath, widely expanded in water.

*Culture characteristics*: Colonies on CMD 50-mm diam after 3 months at 22 °C, first hyaline, partly or entirely turning brownish or ochre, covered by floccose tufts or a dense white mat of aerial hyphae, with irregular wavy margin; reverse ochraceous with a caramel to dark brown centre. Conidia not observed in culture.

*Habitat*: on dead corticated branches of *Quercus* spp., constantly associated with effete conidiomata of *Coryneum* spp.

*Distribution*: apparently widely distributed in south-central and Southern Europe; confirmed from eastern Austria, Greece (Crete), Italy and Spain.

*Holotype*: Spain, Jaén, Fuensanta de Martos, Sierra de la Grana, 37° 36′ 18.93″ N, 3° 54′ 55.91″ W, 747 m a.s.l., on dead branch of *Quercus faginea*, 20 Mar. 2018, leg. S. Tello, S.T.20031801 (WU 40027, holomorph; ex-holotype culture CBS 147011 = MYT).

*Additional specimens examined*: Austria, Burgenland, Siegendorf, Siegendorfer Puszta, 47° 46′ 44.2″ N, 16° 34′ 53.7″ E, on dead corticated branches of *Quercus cerris*, 25 Apr. 2020, leg. H. Voglmayr and I. Krisai-Greilhuber (WU 40028; asexual morph only); Niederösterreich, Gießhübl, Predigerstuhlwiese, 48° 06′ 28.8″ N, 16° 12′ 38.3″ E, on dead corticated branches of *Quercus cerris*, 28 May 2017, leg. H. Voglmayr (WU 40029; asexual morph only); Hagenbrunn, Bisamberg, near Zigeunerbründl, 48° 19′ 10.6″ N, 16° 24′ 09.1″ E, on dead corticated branches of *Quercus cerris*, 5 Feb. 2017, leg. H. Voglmayr and W. Jaklitsch (WU 40030; asexual morph only); Wien, Landstraße, Botanical Garden of the University (HBV), 48° 11′ 25.0″ N, 16° 22′ 57.6″ E, on dead corticated branches of *Quercus cerris*, 31 Aug. 2017; ibid., 17 Feb. 2020, leg. H. Voglmayr (WU 40031; asexual morph only); Wien, Hernals, Schwarzenbergpark, northern margin of the Tiefauwiese, 48° 14′ 50.2″ N, 16° 16′ 43.3″ E, on dead corticated branches of *Quercus cerris*, 15 Apr. 2018, leg. H. Voglmayr (WU 40032; asexual morph only); Wien, Ottakring, Wilhelminenberg, Galitzinberg, 48° 13′ 03.9″ N, 16° 16′ 35.8″ E, on dead corticated branches of *Quercus cerris*, 1 Jul. 2017, leg. H. Voglmayr (WU 40033; asexual morph only). Greece, Crete, Rethimno, Prines, 35° 20′ 15.4″ N, 24° 25′ 14.3″ E, on dead corticated branch of *Quercus macrolepis*, 7 Jun. 2015, leg. H. Voglmayr and W. Jaklitsch (WU 40034, culture D135 = CBS 144229; asexual morph only); Armeni, 35° 17′ 51.4″ N, 24° 28′ 06.6″ E, on dead corticated branch of *Quercus macrolepis*, 27 Jun. 2018, leg. W. Jaklitsch (WU 40035; asexual morph only). Italy, Sicilia, Etna, above Linguaglossa, 37° 50′ 20.7″ N, 15° 07′ 01.0″ E, on dead corticated branch of *Quercus pubescens*, 18 Jun. 2016, leg. H. Voglmayr and W. Jaklitsch (WU 40036; asexual morph only). Spain, Asturias, Pola de Somiedo, on dead branches of *Quercus ilex* still attached to the tree, 43° 05′ 49.1″ N, 6° 15′ 17.3″ W, 2 Jun. 2013, leg. H. Voglmayr (WU 40044); ibid., Camino de Pola a Castro, 43° 07′ 12.04″ N, 6° 15′ 15.46″ W, 745 m, on dead branches of *Quercus ilex* still attached to the tree, 8 Jun 2017, leg. S. Tello S.T.08061701; Jaén, Aldeaquemada, Carretera de Aldeaquemada, 30SVH 61841 50351, 38° 24′ 02.05″ N, 3° 26′ 13.23″ W, 975 m, on dead branch of *Quercus suber*, 23 Feb. 2020, leg. S. Tello S.T.23022002 (WU 40037; asexual morph only); Jaén, Andújar, Peñascales, 38° 06′ 46.58″ N, 4° 01′ 29.45″ W, 650 m, on dead branches of *Quercus ilex* attached to the tree, in mixed forest of *Q. ilex* and *Pinus pinea* with *Cistus ladanifer*, 24 Mar. 2016, leg. S. Tello, (WU 40038, JA-CUSSTA 8291; culture EXT1; holomorph); Jaén, Valdepeñas de Jaén, La Solana, 30SVG 29011 60779, 37° 35′ 28.82″ N, 3° 48′ 14.80″ W, 1155 m, on corticated branches of *Quercus ilex*, 12 May 2016, leg. S. Tello, (JA-CUSSTA 8297, WU 40039; holomorph); Jaén, Valdepeñas de Jaén, Humbría de Ventisqueros, 37° 36′ 47.54″ N, 3° 44′ 47.83″ W, 1205 m, on dead branches of *Quercus ilex* still attached to the tree, 13 Aug. 2017, leg. S. Tello S.T.13081703; Jaén, Valdepeñas de Jaén, Puerto de las coberteras, 37° 37′ 19.83″ N, 3° 45′ 44.11″ W, 1334 m, on dead branches of *Quercus ilex* still attached to the tree, 5 Mar. 2017, leg. S. Tello S.T.05031702.

*Notes*: *Mycosphaerangium quercinum* resembles the North American *M. tetrasporum*, which, however, mainly differs by distinctly swollen, clavate, red brown paraphysis tips, lack of croziers at the ascus base and distinctly verruculose ascospore walls. It differs from *M. magnisporum* by distinctly shorter, subglobose to broadly ellipsoid, concolorous ascospores and a different host. In all specimens investigated, conidiomata as well as ascomata of *M. quercinum* are associated with effete conidiomata of *Coryneum* spp., indicating that it is fungicolous. Although *M. quercinum* seems to be widely distributed in Southern Europe, ascomata are so far only known from Spain.

**Fig. 7:**
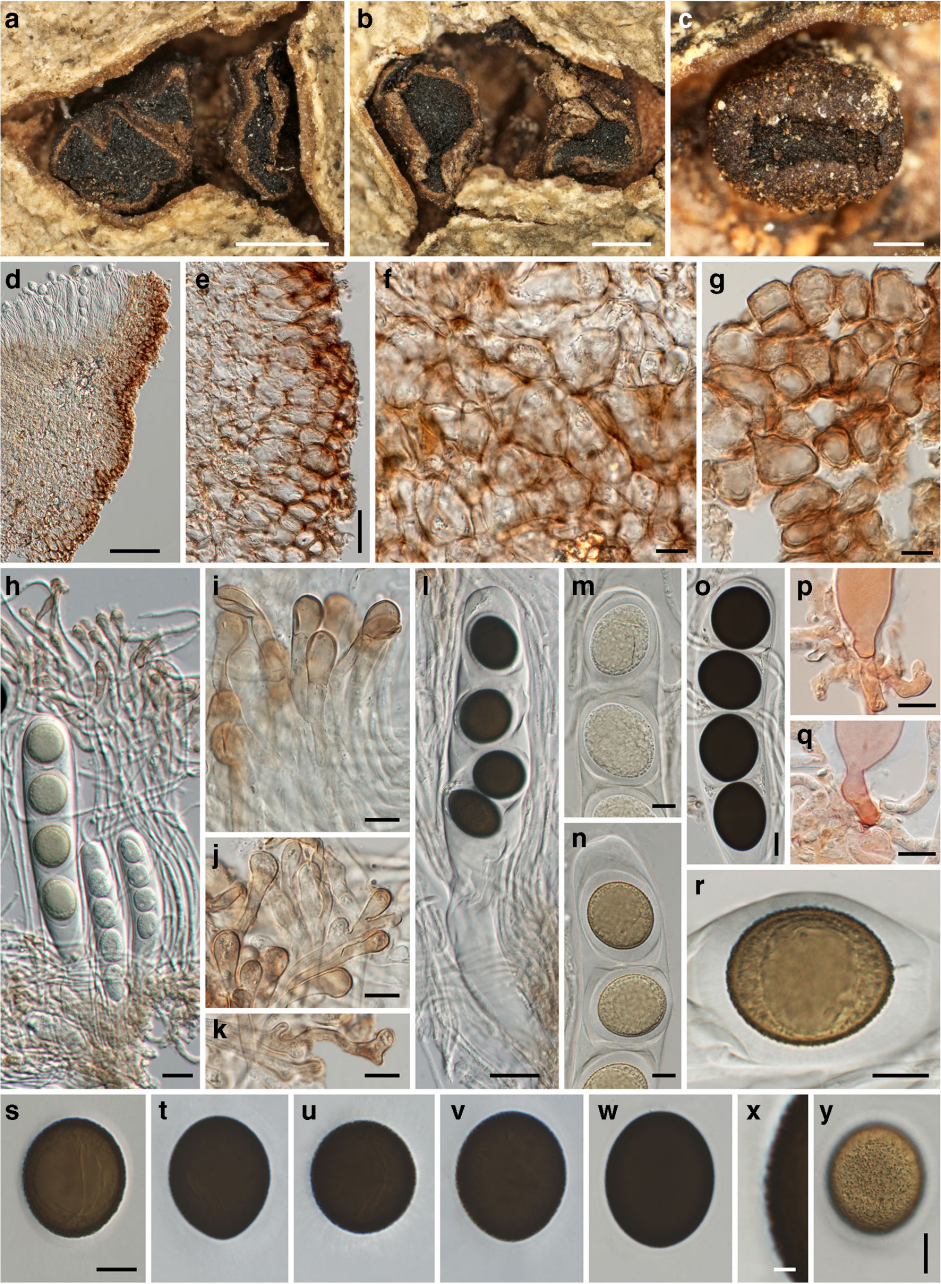
*Mycosphaerangium tetrasporum*, sexual morph ((**a**–**c**, **g**, **i**, **l**–**o**, **q**–**u**, **w**, **x**) GZU 000335639, lectotype; (**d–f, h, j, k, p, v**) WU 40040). (**a–c**) Apothecia in face view. (**d**) Apothecium margin in section. (**e**) Marginal excipulum in section. (**f**) Medulla in section. (**g**) Ectal excipulum cells in squash mount. (**h**) Paraphyses and immature asci. (**i**–**k**) Swollen, reddish brown paraphysis tips. (**l**) Mature ascus with four ascospores surrounded by thick gelatinous sheaths. (**m**–**o**) Ascus apices ((**m, n**) immature). (**p, q**) Ascus bases with simple septa. (**r**–**w**) Ascospores (in (**r**) showing widely expanded gelatinous sheath and distinctly verruculose wall; (**x**, **y**) verruculose contours and wall in face view). All mounts dead and in 3% KOH, except (**p**, **q**) in Congo red. Scale bars: (**a, b**) 500 μm; (**c, d**) 200 μm; (**e**) 30 μm; (**f**–**k**, **m**–**w**, **y**) 10 μm; (**l**) 20 μm; (**x**) 2 μm

**Fig. 8:**
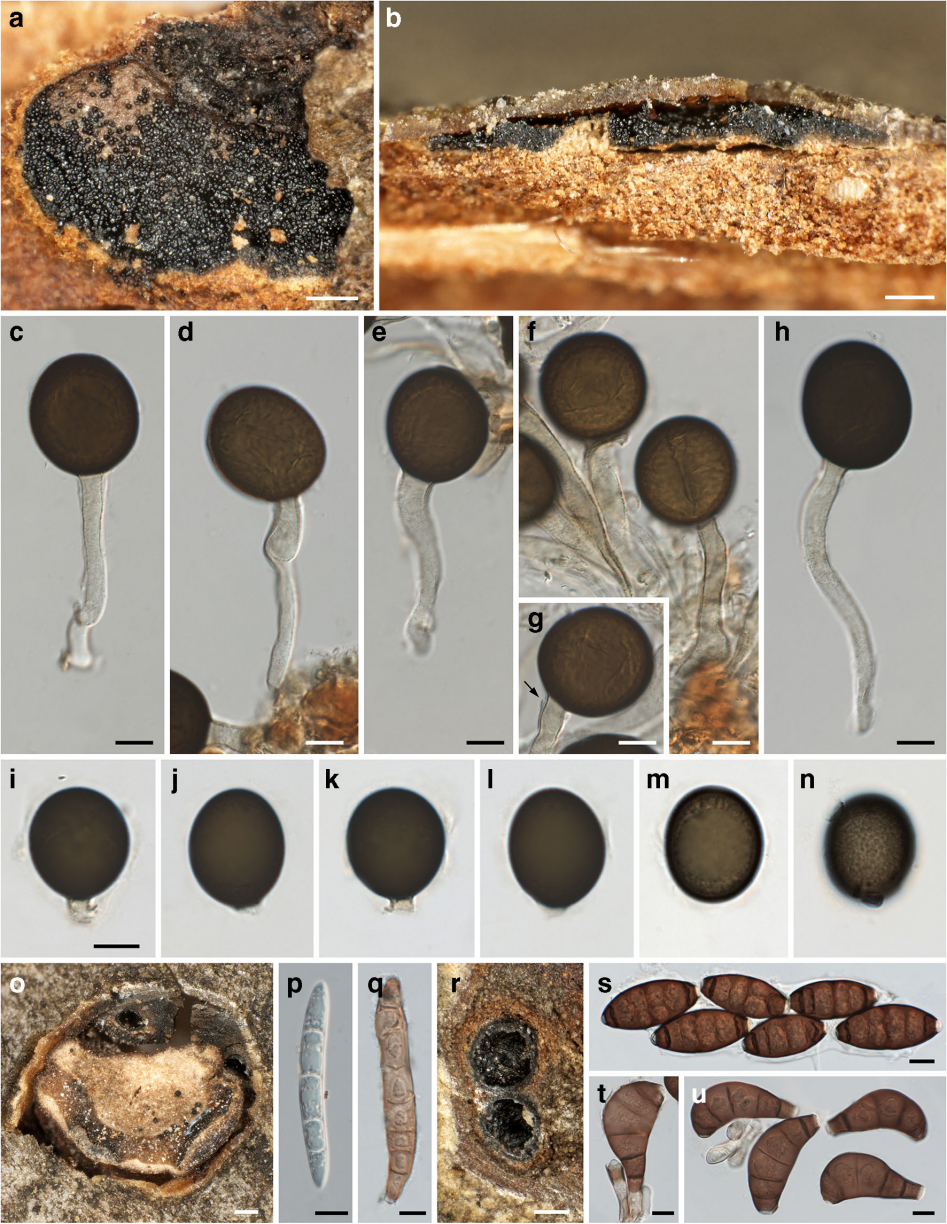
*Mycosphaerangium tetrasporum*, asexual morph ((**a**–**h**) GZU 000335720, isotype; (**i**–**q**) Lavoie; (**r**–**u**) GZU 000335639, lectotype). (**a**) Conidiomata in face view after removal of cortical layer. (**b**) Conidioma in vertical section. (**c**–**h**) Mature conidia on conidiogenous cells; arrow denoting single annellation. (**i**–**n**) Conidia ((**m**) showing large oil drop, (**n**) verrucae on inner wall). (**o**–**u**) Stromata (**o**, **r**), ascospores (**p**, **s**) and conidia (**q**, **t**, **u**) of *Coryneum* spp. associated with *M. tetrasporum*. All mounts in 3% KOH and dead. Scale bars: (**a, b, o, r**) 200 μm; (**c**–**n**, **p**, **q**, **s**–**u**) 10 μm

***Mycosphaerangium tetrasporum*** (Ellis) Verkley, Stud Mycol 44: 156. 1999. Figures 7 and 8.

*Basionym*. *Dermatea tetraspora* Ellis, Bull. Torrey bot. Club 6: 108. 1876.

≡ *Cenangium tetrasporum* (Ellis) Sacc. Syll. fung. 8: 570. 1889.

≡ *Phaeangium tetrasporum* (Ellis) Sacc. & P. Syd., Syll. fung. 16: 765. 1902.

≡ *Sphaerangium tetrasporum* (Ellis) Seaver, North American Cup-fungi, (Inoperculates) (New York): 308. 1951.

*Sexual morph*: *Apothecia* 0.4–2-mm diam, erumpent from bark, solitary or in groups of 2–5, sessile, without a basal stroma, forming circular to irregular plane to concave discs. *Receptacle* dark red-brown and scaly and the raised margin slightly paler. *Hymenium* roughened, dark brown to black. *Ectal excipulum* consisting of a 50–100-μm-thick layer, narrower at the margin, of dark orange brown textura globulosa-angularis, cells (9–)13–23(–28)-μm diam, with walls 1–2 μm thick. *Medullary excipulum* consisting of orange brown textura globulosa-angularis, cells (7–)13–24(–33)-μm diam. *Subhymenium* made of hyaline to pale brown textura intricata of densely interwoven thin-walled hyphae. *Paraphyses* ca. 250–310 μm long, 2.5–4.8 μm wide, cylindrical, hyaline, septate, branched, longer than the asci, with swollen, club-shaped red brown tips 5–11 μm wide, not encrusted. *Asci* 185–265(–305) × (26–)29–38(−42) μm (*n* = 29), cylindrical-clavate, unitunicate, with rounded to subtruncate apex, inamyloid, without visible apical apparatus, containing 4 uniseriately arranged ascospores, base tapering, with simple septum. *Ascus dehiscence* unknown. *Ascospores* (23.5–)26.5–31.5(−34) × (18.5–)23–27(–29) μm, l/w = (1.0–)1.1–1.2(–1.4), Me = 29 × 25 μm, Qe = 1.15 (*n* = 88), globose to ellipsoid, first hyaline, turning pale brown and finally dark brown to nearly black when mature, containing a large globose oil drop, walls verruculose and appearing rough in profile view, with a prominent refractive gelatinous sheath widely expanding in released spores.

*Asexual morph*: *Conidiomata* acervular, subperidermal, scattered, scarcely raising the bark and becoming visible in the dry state by black irregular spots of distinctly glossy conidia and sometimes small bumps in the bark after the discharge of the conidia through bark fissures. *Conidiophores* arising from conidiomatal base, reduced to conidiogenous cells. *Conidiogenous cells* percurrent, cylindrical, filiform, slightly enlarged apically, 30–100 × (6–)6.3–8.3(–9.5) μm, non-septate, hyaline, turning brownish with age; with only a single annellation and conidium on top. *Conidia* (22.5–)26–33 (–35.5) × (20.5–)22.5–30.5(–33) μm, l/w = (1.0–)1.1–1.2(–1.3), Me = 29.5 × 26.5 μm, Qe = 1.13 (*n* = 55), globose to broadly ellipsoid, brown to nearly black, finely verruculose, surrounded by a gelatinous sheath widely expanding in water.

*Habitat*: on dead corticated branches of *Quercus* spp. (*Q. coccinea*, *Q. rubra*), constantly associated with effete conidiomata or stromata of *Coryneum* spp.

*Distribution*: Eastern North America (USA, Canada).

*Typification*: USA, NJ, Newfield, on dead limbs of *Quercus coccinea* still attached to the tree, March (without day and year), J.B. Ellis s.n., distributed in Ellis. N. Am. Fungi 70 (GZU 000335639, lectotype here designated; MBT393681; isotype GZU 000335720; holomorph).

*Additional specimens examined*: Canada, Québec, Montérégie, on dead corticated branches of *Quercus rubra*, Apr. 2014, A. Lavoie (WU 40040).

*Notes*: *Mycosphaerangium tetrasporum* resembles the European *M. quercinum*, which, however, differs by irregularly sinuous-knobby hyaline paraphysis tips encrusted by amorphous brown plaques, the presence of croziers at the ascus base, and by finely verruculose spores appearing smooth in profile view. It differs from *M. magnisporum* by distinctly shorter, subglobose to broadly ellipsoid, concolorous ascospores and a different host. In all specimens investigated, conidiomata as well as ascomata of *M. tetrasporum* are associated with effete conidiomata or stromata of *Coryneum* species. To our knowledge, besides the type specimen from Newfield, NY (USA), only a recent Canadian collection is known (see above), which unfortunately is scant and in poor condition, and therefore no DNA data could be generated.

***Neomelanconium*** Petr., Annls mycol. 38(2–4): 208. 1940, emend. Voglmayr.

*Type species*: *Neomelanconium gelatosporum* (H. Zimm.) Petr.

*Sexual morph* apothecial, circular to polyangular, erumpent through bark, concave to plane, solitary or in groups, sessile, rough, disk black, basal stroma absent. *Receptacle* whitish, pulverulent, with an external layer of crystals, with incurved, inversely stellate marginal teeth. *Paraphyses* embedded in hymenial gel, slightly longer than the asci, filiform, hyaline, septate, simple or branched, sometimes anastomosing, with hyaline to brownish slightly swollen tips. *Asci* cylindrical-clavate, thin-walled, unitunicate, with broadly rounded apex without a distinct apical apparatus, with 8 uniseriately arranged ascospores. *Ascospores* dark brown, aseptate, subglobose to ellipsoid, with a thick, dark brown, minutely warted wall, surrounded by a prominent hyaline gelatinous sheath.

*Asexual morph* acervular, often conspicuous due to black, glossy, effused conidial masses. *Conidiomata* subperidermal, not to slightly elevating the bark, solitary, scattered, lenticular, with thin, whitish pseudoparenchymatous base, exuding conidia in a black mucoid droplets. *Conidiophores* emerging from a more or less pseudoparenchymatous base, reduced to the conidiogenous cells, hyaline, smooth, cylindrical. *Conidiogenous cells* percurrent, hyaline, usually with a single, rarely few, annellations. *Conidia* dark brown, aseptate, globose, ellipsoid, ovoid, pyriform to clavate, apex broadly rounded, base truncate, thick-walled, guttulate, usually with a large and numerous small guttules, with finely verruculose wall, surrounded by a prominent hyaline gelatinous sheath expanding in water.

*Notes*: No sexual morph has been known for *Neomelanconium* until this study, and the connection with the mycosphaerangium-like sexual morph is confirmed by morphological data. *Neomelanconium* differs from the closely related *Mycosphaerangium* mainly by eight-spored asci and marginal, inversely stellate excipular teeth with a textura prismatica-porrecta covered by an external layer of white crystals. In addition, unlike in *Mycosphaerangium*, there is no evidence for association with fungi in *Neomelanconium*.

Morphology of the second species, *N. deightonii*, fits the generic type, and it is therefore retained within the genus, albeit no sequence data are available to verify its phylogenetic affiliation with *Neomelanconium*. Based on the lack of a gel sheath around the conidia, Crous et al. (2019) transferred the third species of the genus, *N. spartii*, to *Pseudomelanconium*.

**Fig. 9:**
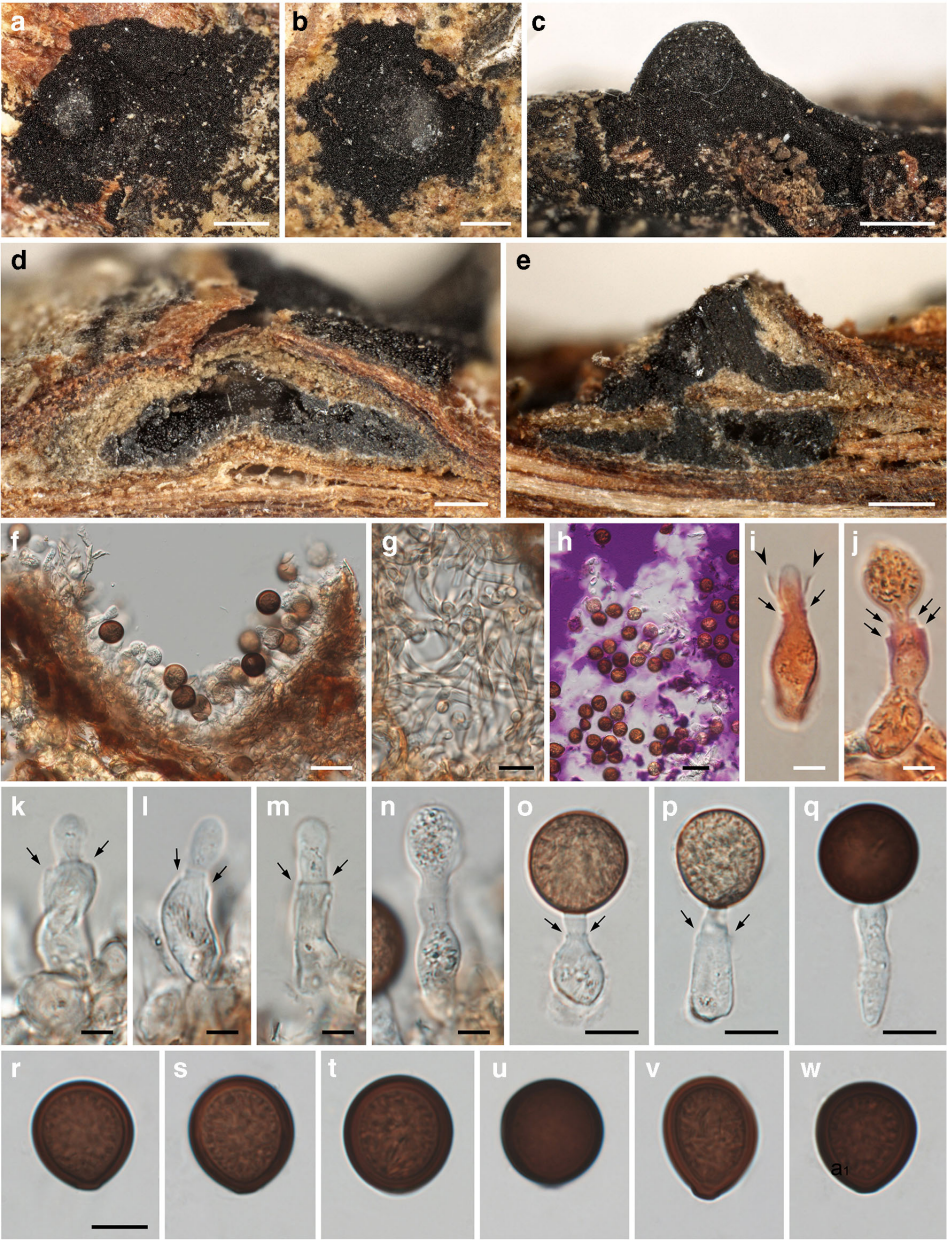
*Neomelanconium deightonii*, asexual morph ((**a**–**d**) W 0102726, lectotype; (**e**–**w**) W 0102724, isotype). (**a**, **b**) Conidiomata with dispersed, glossy conidia in face view. (**c**) Conidioma in lateral view. (**d**–**f**) Conidiomata in vertical section. (**g**) Hyphae of conidiomatal base. (**h**) Conidia in Indian ink showing the gelatinous matrix. (**i**–**q**) Immature (**i**–**p**) and mature (**q**) conidia on conidiogenous cells; arrows and arrowheads denoting annellations and remnants of gelatinous sheath of conidium, respectively. (**r**–**w**) Conidia. All mounts dead and in 3% KOH, except (**h**) in Indian ink and (**i**, **j**) in Congo red. Scale bars: (**a**–**c**) 500 μm; (**d**, **e**) 200 μm; (**f**, **h**) 30 μm; (**g**, **o**–**w**) 10 μm; (**i**–**n**) 5 μm

***Neomelanconium deightonii*** Petr., Sydowia 8(1–6): 51. 1954. Figure 9.

*Sexual morph*: not known.

*Asexual morph*: *Conidiomata* acervular, plurilocular, subperidermal, scattered, raising the bark, at maturity covered by conspicuous black, irregular spots of glossy conidia. *Conidiophores* arising from a brown conidiomatal base, reduced to conidiogenous cells. *Conidiogenous cells* percurrent, cylindrical to ampulliform, (16–)19–28(–38) μm × (4.7–)5.5–8(–9.5) μm (*n* = 30), non-septate, hyaline, at base turning brownish with age, smooth; with 1(–3) annellations and a single conidium on top. *Conidia* (18.5–)18.7–20.5(–22.2) × (17–)18–19.3(–20) μm, l/w = (1–)1–1.1(–1.3), Me = 19.7 × 18.6 μm, Qe = 1.06 (n = 30), globose to ellipsoid, dark brown, surrounded by a gelatinous sheath rapidly dissolving in water.

*Habitat*: on dead corticated branches of *Spondias mombin* (Anacardiaceae).

*Distribution*: known only from the type locality in Sierra Leone (Africa).

*Typification*: Sierra Leone, Njala Kori, in bark of dead branches of *Spondias mombin*, 28 Jan. 1953, F.C. Deighton M 8099 (W 0102726, lectotype here designated; MBT393682; isotypes W 0102724, W 0102725).

*Notes*: The conidiomata, conidiogenous cells and conidia of *Neomelanconium deightonii* resemble those of the generic type, *N. gelatosporum*. As already mentioned by Petrak (1954), *N. deightonii* differs from *N. gelatosporum* mainly by irregularly plurilocular conidiomata, which led him to propose the subgenus *Neomelanconiopsis* for it. Although no sequence data are available, for the time being, we decide to maintain *N. deightonii* within *Neomelanconium* based on morphological resemblance.

There is some confusion in the literature about the gel sheath surrounding the conidia. While Petrak (1954) described a rapidly dissolving gel sheath 1.5–2.5 μm wide, Sutton (1980) reported the absence of a gelatinous sheath. Re-investigations of the original collections showed that within conidiomata, the conidia were embedded in an extensive amorphous gel matrix (Fig. 9h) that apparently originated from dissolved conidial gel sheaths, as remnants were seen on some conidiophores (Fig. 9j). With the evidence at hand, we consider Petrak’s (1954) observations to be correct; a gel sheath surrounding the conidia may be only observed in fresh material, becoming confluent and agglutinated to an amorphous matrix after prolonged storage.

Three isotype collections are present in W, of which W 0102726 is here selected as lectotype based on abundance.

**Fig. 10:**
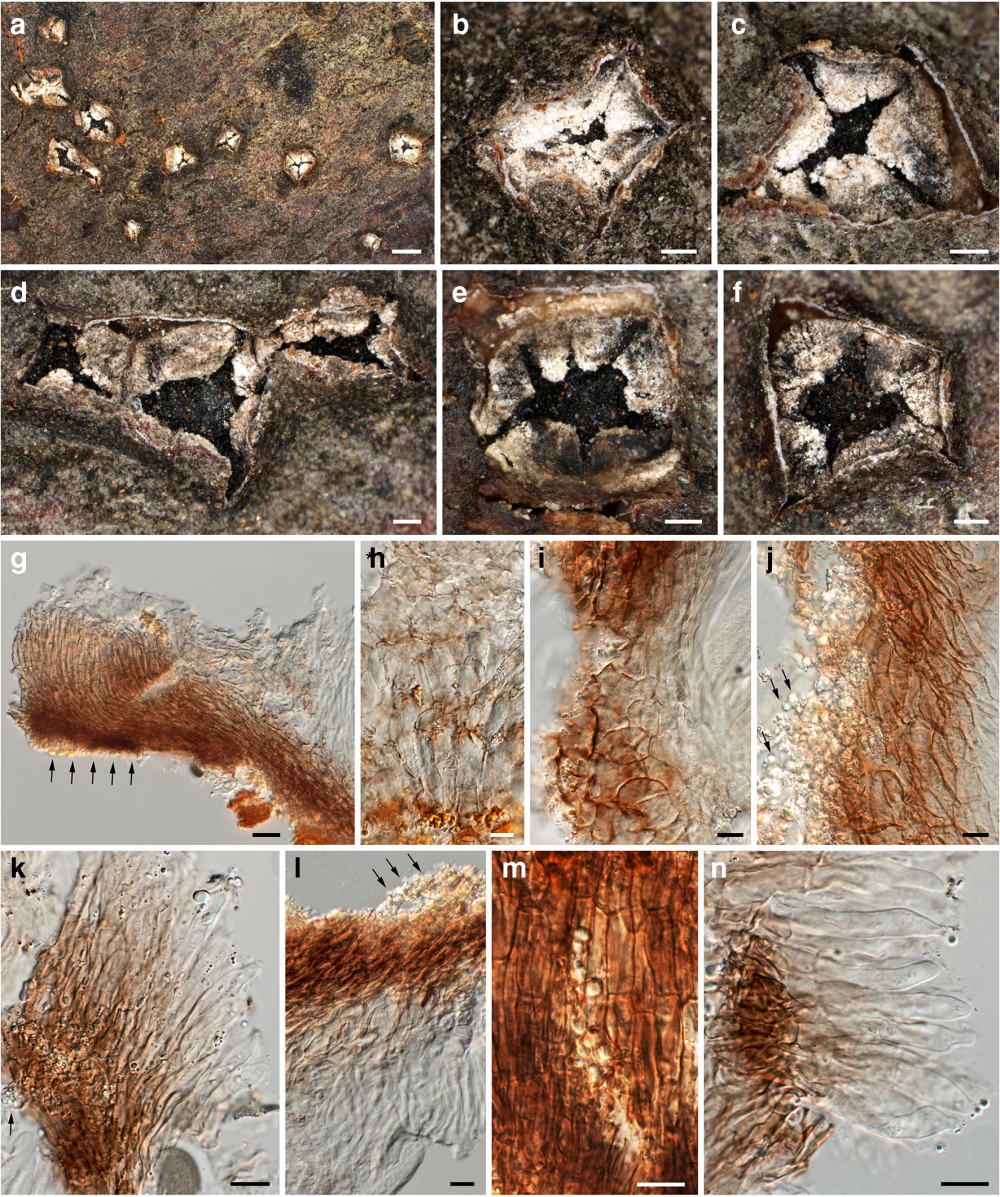
*Neomelanconium gelatosporum*, sexual morph (NY 02792207, lectotype of *Sphaerangium tiliae*). (**a**–**f**) Apothecia in face view. (**g**) Ectal excipulum and excipulum teeth in section; arrows denoting external crystals. (**h**) Medullary excipulum and subhymenium in section. (**i**) Ectal excipulum in section at base. (**j**–**l**) Excipulum teeth in section; arrows denoting external crystals. (**m**) Excipulum teeth in squash mount, showing textura prismatica-porrecta. (**n**) Hyaline, elongate, apically rounded end cells of excipulum teeth. All mounts in 3% KOH and dead. Scale bars: (**a**) 1 mm; (**b**–**f**) 200 μm; (**g**) 20 μm; (**h**–**n**) 10 μm

**Fig. 11:**
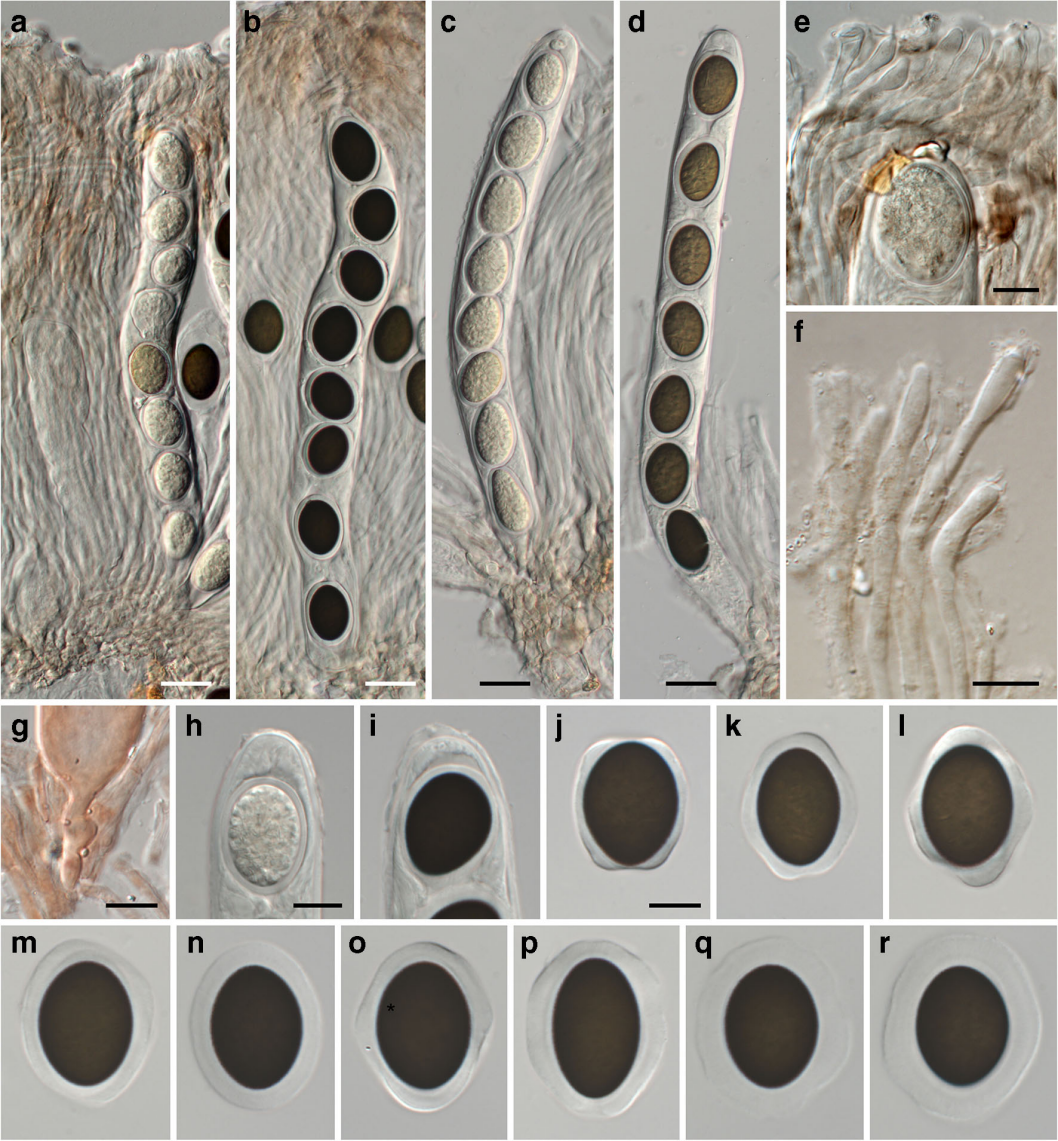
*Neomelanconium gelatosporum*, sexual morph (NY 02792207, lectotype of *Sphaerangium tiliae*). (**a**–**d**) Asci ((**a**, **c**) immature, (**b**, **d**) mature) and paraphyses. (**e**, **f**) Apically swollen paraphysis tips embedded in pale brownish gelatinous matrix and ascus apex (**e**). (**g**) Ascus base with simple septum. (**h**, **i**) Ascus apices ((**h**) immature, (**i**) mature). (**j**–**r**) Ascospores. All in 3% KOH and dead. Scale bars: (**a**–**d**) 20 μm, (**e**–**r**) 10 μm

**Fig. 12:**
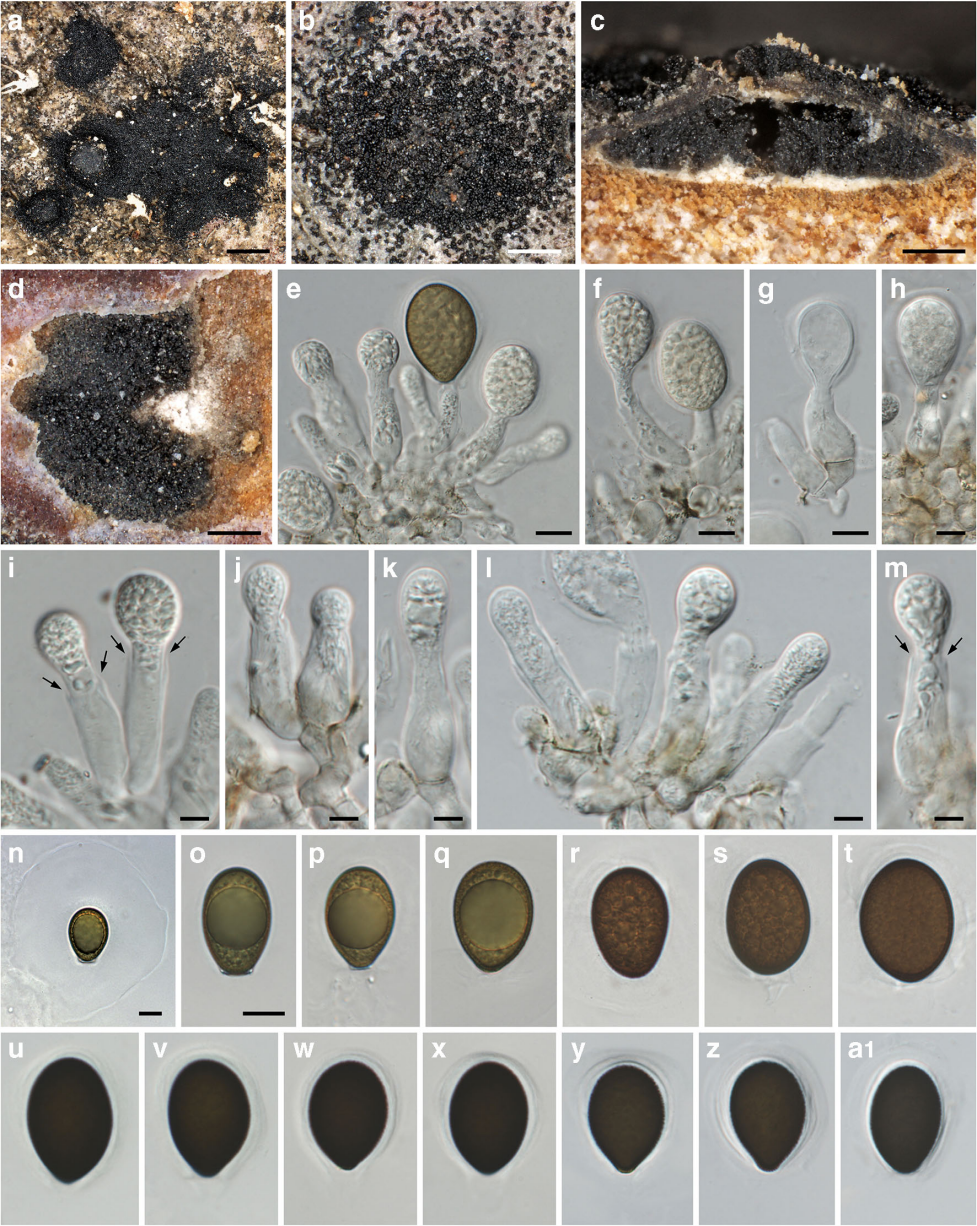
*Neomelanconium gelatosporum*, asexual morph ((**a**, **c**–**f**, **i**, **u**–**x**) W 1978-11035, isotype of *Melanconium gelatosporum*; (**b**, **g**, **h**, **j**–**m**, **y**–**a1**) NY 02792207, lectotype of *Sphaerangium tiliae*; (**n**–**q**) WU 40042; (**r**–**t**) WU 40041). (**a**, **b**) Conidiomata with dispersed, glossy conidia in face view. (**c**) Conidioma in vertical section. (**d**) Conidioma in transverse section. (**e**–**m**) Immature (**e**–**m**) and partly mature (**e**) conidia on conidiogenous cells; arrows denoting single annellations. (**n**–**a1**) Conidia ((**n**) with widely expanded gelatinous sheath). All in water; (**n**–**t**) vital, all others dead. Scale bars: (**a**) 500 mm; (**b**–**d**) 200 μm; (**e**–**g**, **n**–**a1**) 10 μm, (**h**–**m**) 5 μm

***Neomelanconium gelatosporum*** (H. Zimm.) Petr., Annls mycol. 38(2–4): 209. 1940. Figures 10, 11 and 12.

*Basionym*. *Melanconium gelatosporum* H. Zimm., Verh. nat. Ver. Brünn 52: 111. 1914.

= *Sphaerangium tiliae* Seaver, North American Cup-fungi, (Inoperculates) (New York): 309. 1951.

≡ *Mycosphaerangium tiliae* (Seaver) Verkley, Stud. Mycol. 44: 157. 1999.

*Sexual morph*: *Apothecia* 0.5–1.2(–2)-mm diam (*n* = 27), erumpent from bark, solitary, sessile, without a basal stroma, broadly adnate to the substrate, first closed, then opening with more or less irregular, inversely stellate whitish teeth 200–440 μm long and 170–800 μm broad, exposing the circular to polyangular, plane, blackish hymenium. *Receptacle* and margin coated by whitish, pulverulent amorphous crystals. *Ectal excipulum* red-brown, consisting of a ca. forty- to 100-μm-thick layer, towards apothecium base of a textura globulosa-angularis of thin-walled cells 12–23 × 6–11 μm, at margin teeth with a textura prismatica-porrecta of concolorous, thin-walled cells 10–25(–34) × 3–8 μm, teeth cells close to the hymenium hyaline, elongate, apically rounded 4–8 μm wide. *Medullary excipulum* consisting of reddish to light brown textura angularis of thin-walled cells (8–)13–31(–40) × (6.5–)8.5–15(–19.5) μm. *Subhymenium* made of subhyaline to brownish textura angularis of thin-walled cells 7–16-μm diam. *Paraphyses* ca. 270–330 μm long, 2–3 μm wide, cylindrical, hyaline, septate, branched, longer than the asci, with slightly swollen, club-shaped tips 3–6 μm wide, not encrusted but embedded in a brownish, granular, gelatinous matrix. *Asci* (250–)260–300(–310) × (23–)23.5–26.5(–27.5) μm (*n* = 10), cylindrical, unitunicate, with rounded apex, inamyloid, without visible apical apparatus, containing 8 uniseriately arranged ascospores, base abruptly tapering, with simple septum. *Ascus dehiscence* unknown. *Ascospores* (18–)22–26 (–27) × (15–)16.5–18.5(–19.5) μm, l/w = (1.2–)1.3–1.5(–1.7), Me = 24.1 × 17.5 μm, Qe = 1.38 (*n* = 66), ellipsoid, multiguttulate, first hyaline, turning pale brown and finally dark brown to nearly black when mature, wall finely verruculose appearing smooth to finely roughend in profile view, with a prominent refractive gelatinous sheath widely expanded in released spores.

*Asexual morph*: *Conidiomata* acervular, subperidermal, lenticular, scattered, scarcely raising the bark and becoming visible in the dry state by black irregular spots of distinctly glossy conidia and sometimes distinct bumps in the bark after the discharge of the conidia through bark fissures. *Conidiophores* arising from a white to cream conidiomatal base, reduced to conidiogenous cells. *Conidiogenous cells* percurrent, cylindrical to ampulliform, (18.7–)21.5–30(–34.5) μm × (6.2–)7.7–10(–11) μm (*n* = 42), non-septate, hyaline, at base turning brownish with age, smooth; with usually a single annellation and conidium on top. *Conidia* (21.5–)24.5–28(–32.5) × (14–)17–19.5(–21) μm, l/w = (1.2–)1.3–1.6(–2), Me = 26.3 × 18.2 μm, Qe = 1.45 (*n* = 260), globose to ellipsoid, olive brown to nearly black, containing a large globose oil drop and numerous small guttules, with finely verruculose wall surrounded by a gelatinous sheath widely expanding in water.

*Culture characteristics*: Colonies on CMD 45-mm diam after 2 months at 22 °C, first cream, becoming yellowish with brownish spots in the centre, surface shining, without aerial hyphae, with regular margin; reverse yellow with a darker centre. Conidia not observed in culture.

*Habitat*: on dead corticated branches of *Tilia* spp.

*Distribution*: Europe (known from Austria, Czech Republic, Germany) and North America (eastern USA).

*Typification*: Czech Republic, Morava, Lednice (formerly Eisgrub), Unterwald, on dead stems and branches of *Tilia* sp., 15 Jan. 1913, H. Zimmermann (W 1978-11035, isotype of *Melanconium gelatosporum*). USA, GA, Clarke, Athens, University of Georgia, Agricultural Campus, in bark of *Tilia americana *var*. heterophylla*, 15 Feb. 1934, J.H. Miller (NY 02792207, lectotype of *Sphaerangium tiliae* here designated; MBT393939; isotype GAM00009299; holomorph).

*Additional specimens examined*: Austria, Niederösterreich, Mayerling, Helenental, 48° 02′ 15.2″ N, 16° 06′ 33.8″ E, on corticated fallen branches of *Tilia* sp., 8 Dec. 2016, H. Voglmayr (WU 40041; culture NG = CBS 143625); Oberösterreich, Raab, Wetzlbach, 48° 21′ 28.7″ N, 13° 40′ 15.7″ E, on corticated fallen branches of *Tilia cordata*, 28 Dec. 2016, H. Voglmayr (WU 40042; culture NG1 = CBS 143626); St. Willibald, Große Sallet, riverine forest SE Loitzmayr, 48° 20′ 42.9″ N, 13° 42′ 59.6″ E, on corticated fallen branches of *Tilia cordata*, 12 Apr. 2020, H. Voglmayr (WU 40043). USA, GA, Clarke, Athens, University of Georgia, Agricultural Campus, in bark of *Tilia americana *var*. heterophylla*, 17 Dec. 1940, G.E. Thompson and J.H. Miller (GAM 00010426, holomorph).

*Notes*: *Neomelanconium gelatosporum* has been described as asexual morph from Europe, and no sexual morph is yet known from Europe. The type specimen of the North American *Mycosphaerangium tiliae*, here considered to be a synonym of *N. gelatosporum*, contains the holomorph. No sequence data are available for *M. tiliae*, but its asexual morph tightly associated with apothecia is morphologically indistinguishable from the European *N. gelatosporum*, indicating conspecificity of the European and North American collections.

Crous et al. (2019) lectotypified the species with a copy of the exsiccatum preserved in H, which they epitypified with a recent collection from Germany for which sequences were generated. Our sequences are identical with those of Crous et al. (2019), and we here add an emended description based on own observations of sexual and asexual morphs, complemented with published descriptions. Conversely to Crous et al. (2019) who observed macro- and microconidia in pure culture, no asexual morph was formed in our cultures.

The original description of *Sphaerangium tiliae* by Seaver (1941) is fragmentary, as it is mostly confined to macroscopic observations. Apart from paraphyses, asci and ascospores, no microscopic data were provided for important apothecial features like excipulum and subhymenium. Although the lectotype collection of *Mycosphaerangium tiliae* is abundant, the investigations were hampered by the very brittle apothecia which precluded preparing entire apothecial sections, and therefore microscopic investigations were kept to a minimum to preserve the type. Thus, only section fragments and squash mounts could be microscopically observed and illustrated, especially for the very delicate excipulum teeth.

## Discussion

Until our investigations, the phylogenetic affiliation of the genus *Mycosphaerangium* within Helotiales remained unclear, and no asexual morph was known. Based on our morphological and molecular phylogenetic data, a close relationship of the genera *Mycosphaerangium* and *Neomelanconium* is here conclusively established. This is in line with morphological similarities of their sexual and asexual morphs, as both ascospores and conidia have a similar size, shape, colour and, most conspicuously, a prominent, widely expanding gel sheath. However, the genus *Neomelanconium*, for which a connection with a sexual morph is here provided for the first time, differs from *Mycosphaerangium* in several respects (e.g. 8-spored asci, inversely stellate, white excipulum teeth with a different tissue type). Therefore, the genera *Mycosphaerangium* and *Neomelanconium* are here recognized as distinct albeit closely related genera. Although no sequence data are yet available for the North American *M. tetrasporum* and *M. magnisporum*, their morphology and ecology leave no doubts that they are closely related to the new European species *M. quercinum*, for which molecular data of both morphs are available.

Also ecologically, there are differences between the genera *Mycosphaerangium* and *Neomelanconium*. For the first time, we here report a close, regular association of all *Mycosphaerangium* species with effete fructifications of the diaporthalean genus *Coryneum*, indicating a fungicolous habit of *Mycosphaerangium*. However, it is currently unclear whether this relation with *Coryneum* is parasitic or necrotrophic. No such association with other fungi was evident in *Neomelanconium* species, which appear to be saprotrophic on their host plants.

Remarkably, no sexual morph has been found for *Neomelanconium gelatosporum* in Europe. However, this may be due to climatic reasons; likewise, also for the newly described *M. quercinum*, no sexual morph is known from Central European accessions despite thorough searches for several years, while it has been repeatedly found in Spain. Therefore, it may be worth searching for the sexual morph of *N. gelatosporum* in warmer, more southern latitudes.

As pointed out by Pärtel and Põldmaa (2019), the Cenangiaceae are a morphologically diverse and heterogeneous assemblage, both in their sexual and asexual morphs. With the inclusion of Hemiphacidiaceae, the extended Cenangiaceae contains members with various ascomatal morphology, ranging from hemiphacidioid, reduced apothecia submerged in the substrate to erumpent, cenangioid, cupulate, rarely even capitate, apothecia (Pärtel et al. 2017). While shape and size of the ascomata is variable between genera, most representatives share apothecia which are closed in young stages or in dry periods, either by a lid or a roof-like apothecial margin (Pärtel et al. 2017). In micromorphology, representatives are typically characterized by *Calycina*-like, usually amyloid apical ascus rings, hyaline (rarely brown) unicellular ascospores and frequent occurrenc of refractive vacuolar bodies (VBs) in the paraphyses or the excipular cells (Jaklitsch et al. 2016, Pärtel et al. 2017).

Asexual morphs in Cenangiaceae are poorly known and documented (Pärtel and Põldmaa 2019). Where known, they are morphologically diverse, being acervular, sporodochial or stromatic with various conidial types, ranging from multicellular stauroconidia to peanut-shaped, allantoid or cylindrical, unicellular conidia with or without appendages (Pärtel et al. 2017, Pärtel and Põldmaa 2019). However, acropleurogenous conidiogeneous cells with conidia developing apically and along the sides were considered to be a common character for Cenangiaceae (Pärtel and Põldmaa 2019).

By their dark brown verruculose ascospores and conidia surrounded by a massive gel sheath, the lack of a visible apical apparatus and, at least in *M. quercinum*, of vacuolar bodies (VBs), the genera *Mycosphaerangium* and *Neomelanconium* deviate significantly from other Cenangiaceae, widening its morphological diversity. Based on the deviating ascal and ascospore features, Verkley (1999) even speculated about a closer affinity of *Mycosphaerangium* with Pezizales, but he could not determine the dehiscence mechanisms in the old herbarium material he had at hand for investigation. However, the fresh collections of the newly described *M. quercinum* indicate dehiscence by a rupture of the ascus apex.

Remarkably, *Mycosphaerangium* and *Neomelanconium* show some similarities to *Cenangium ferruginosum*, which also has erumpent apothecia, inamyloid asci, and ascospores with a gel sheath. In addition, the VBs that are characteristic of many members of the family are absent in *Cenangium* and in *Mycosphaerangium*. However, in the phylogenetic analyses, *Cenangium* is not closely related to *Mycosphaerangium* and *Neomelanconium*, indicating an independent evolutionary origin of these similarities.

Ecologically, *Mycosphaerangium* and *Neomelanconium* fit well the Cenangiaceae by growth on corticated branches still attached to the trees, which requires adaptations to drought. Colonization of aerial host parts (twigs or leaves) is a common ecological character of Cenangiaceae, which is connected with longevity and drought resistance of their apothecia (Pärtel et al. 2017). Therefore, morphological characters like rupture of the ascus apex and thick-walled, dark brown ascospores and conidia surrounded by a thick gel sheath may represent an adaptation to the dry conditions in their habitats.
